# P2P lending platforms in Malaysia: What do we know?

**DOI:** 10.12688/f1000research.73410.1

**Published:** 2021-10-26

**Authors:** Lan Thi Phuong Nguyen, Wisdom Kalabeke, Saravanan Muthaiyah, Ming Yu Cheng, Kwan Jing Hui, Hazik Mohamed

**Affiliations:** 1 FACULTY OF MANAGEMENT, MULTIMEDIA UNIVERSITY, CYBERJAYA, SELANGOR, 63100, Malaysia; 2 Faculty of Accountancy and Management, Universiti Tunku Abdul Rahman, Bandar Sungai Long Cheras, Kajang, Selangor, 43000, Malaysia; 3 Stella Consulting, Stella Consulting, 60 Paya Lebar Square #07-44,, 409051, Singapore

**Keywords:** P2P lending platforms, default risk, SMEs, individual investors, credit rating, FinTech platforms

## Abstract

**Background** - With the recent evolution of Financial Technology (FinTech), 11 peers to peer (P2P) lending platforms have been regulated by the Securities Commission in Malaysia since 2016. P2P lending platforms offer new investment opportunities to individual investors to earn higher rates on return than what traditional lenders usually provide. However, individual investors may face higher potential risks of default from their borrowers. Therefore, individual investors need to understand the potential exposure to such P2P lending platforms to make an effective investment decision. This study aims to explore the potential risk exposures that individual investors may experience at Malaysia's licensed P2P lending platforms.

**Methods** - Based on data collected manually from nine P2P lending platforms over five months, relationships between interest rates and various risk classifying factors such as credit rating, industry, business stage, loan purpose, and loan duration are examined.

** Results**- This study shows that loans with a similar credit rating and with or without similar loan purpose; and a business stage may offer investors significantly different interest rates. In addition, loans with shorter durations may provide investors with higher interest rates than those with longer durations. Finally, loans issued by companies from the same industry appeared to be charged with similar interest. These findings are valuable to investors to prepare themselves before making their investments at the P2P lending platforms.

**Conclusion**- With first hand-collected data, this study provides an original insight into Malaysia's current P2P lending platforms. Findings obtained for relationships between interest rates and risk classifying factors such as credit rating, industry, business stage, loan purpose and loan duration are valuable to investors of Malaysian P2P lending platforms.

## Introduction

As the most prominent sector in Financial Technology (FinTech), peer-to-peer (P2P) lending platforms use digital communication technology to connect lenders and borrowers online. FinTech platforms like P2P lending are easily accessed by smartphones and the internet for getting unconventional loans. Unlike traditional banks, these lending platforms do not require collateral, the ownership of a bank account, and a business plan. Borrowers are rated and categorized based on their risk profile determined by their provided personal information, including information from their social media sites.

Since the first two P2P lending platforms, named “Zopa” and “Prosper”, were launched in the U.K. and U.S. in 2005 and 2006, respectively, the P2P lending industry has grown and reached Asia. The credit crunch experienced by many traditional banks in the U.S. because of the 2008 Global Financial Crisis, forced many borrowers to turn to P2P lending platforms for their short-term financing needs. Business Wire (2020) reported that the annual compounded growth rate of the global peer to peer (P2P) lending market was around 25% between 2014 and 2019. Although there has been a slowdown in FinTech investments across America and Europe since 2016, the number of P2P lending platforms continues to increase in many emerging markets in the past few years ( Stern, Makinen, and Qian, 2017).

Since 2016, 11 peer-to-peer (P2P) lending platforms have been granted licenses by the Securities Commission in Malaysia. Since then, the industry has experienced healthy growth in its first two and a half years of operation, as reported by Zhe (2019). Within the first nine months of operation, RM521.7 million were raised by the first six licensed P2P lending platforms to fund 1,560 small and medium enterprises (SMEs). By the end of 2019, the numbers of registered investors at the three most active platforms, Funding Societies Malaysia, Fundaztic and B2B FinPal, were 3,500, 1,700, and 1,500, respectively.

Kok (2021) reposted that many P2P lending platforms, i.e., Funding Societies Malaysia and Fundaztic, experienced steady growth in 2020 despite the Covid-19 pandemic. Their default rates were reported to be reasonably low at 3% and 2.5% per annum by Funding Societies and Fundaztic, respectively. There are two main reasons for this strong growth of P2P lending platforms: the cut in interest rates by Bank Negara Malaysia from 3% to 1.75% between December 2019 and July 2020, and the growth in the use of digital space during the pandemic. Thus, P2P lending platforms appear to be attractive alternative investment platforms as they offer much higher interest rates, ranging from 6% to 21% per annum across the eleven platforms.

Unlike P2P lending platforms in the West, P2P lending platforms in Malaysia are designed to give out loans only to businesses and not individual borrowers. For SMEs that cannot provide sound evidence on their business performance and enough collateral, P2P lending platforms are their best financing alternatives. However, this could imply a possibly higher rate of default faced by the P2P lending industry in Malaysia than traditional lending institutions. Therefore, investors are often advised to diversify their funds across different investment notes while investing at each platform. However, having an effective diversification strategy depends heavily on two critical elements: (1) whether investors have basic financial literacy and (2) how much information of each investment note is made known to investors by each P2P platform.

When it comes to evaluating the default risk of a borrower, information that can help arrive at one's estimated credit score is needed. For P2P lending platforms, the borrowers' credit score is based solely on information supplied by the borrower ( Li, 2018). Since the information provided by P2P lending platforms are limited and not guaranteed, it would be even more challenging for investors with good financial literacy to reach a sound investment decision. Therefore, individual investors need to understand the potential exposure to such P2P lending platforms to be able to make an effective investment decision. This study explores potential exposures that individual investors may experience at licensed P2P lending platforms in Malaysia.

## Methods

Individual investors would mainly like to subscribe to platforms with affordable subscription fees. Of 11 licensed P2P lending platforms in Malaysia, only 10 platforms (Funding Societies, B2B FinPal, Fundaztic, QuicKash, AlixCo, Nusa Kapital, CapSphere, MicroLEAP, Cofundr, and Money Save) require affordable initial deposits, ranging from RM5 to RM1,000, from individual investors. CapBay was excluded from this study as it requires the highest amount of initial deposit (RM10,000) from its investors. Additionally, Funding Societies was also not considered due to the complexity of its subscription. Therefore, the final sample size was nine P2P lending platforms (B2B, Fundaztic, QuicKash, AlixCo, Nusa Kapital, CapSphere, MicroLEAP, Cofundr, and Money Save).

All available information was obtained from the nine platforms for comparative analysis ( Underlying data) ( Nguyen, 2021). The data collection period was from January to May 2021. Data collection was done manually as a loan campaign is only announced to investors for a maximum of seven days at each platform. Once a campaign is bided on successfully, the issue note will be taken down, and its record will no longer be visible to investors who did not invest in that note.

The lender decides whom to grant the loan primarily based on the interest rate. The percentage of the interest rate is mainly dependent on the borrower’s credit score, which is based on various loan characteristics such as the type of business, business cycle, loan purpose, loan duration, and loan amount. Therefore, understanding the relationship between the prime factor - interest rate and other secondary factors such as credit score, loan term, loan duration, loan purpose, business cycle and industry will help investors to be more aware of what to expect when making their investment decisions at those platforms.

### Statistical analysis

Since there is no data publicly available for individual P2P lending platforms in Malaysia, this study employs first hand-collected information for the analysis. Descriptive and cross-tabulation statistics were produced by Microsoft Excel 2010 and IBM SPSS Statistics 26, using a five-month data set to examine the potential exposure that individual investors may face at the nine selected P2P lending platforms. In addition, other tests for normality, coefficients, and multiple regression tests were also conducted. However, only descriptive statistics are reported in this paper.

## Results/discussion

After the collapse of hundreds of P2P lending platforms in China since 2013 ( Bloomberg Businessweek, October 3, 2018) due to fraudulent activities, P2P lending in Malaysia was somewhat restrictive until 2016. Malaysia is the first country in Southeast Asia to regulate its first six P2P market operators in 2016 (The Securities Commission, 2017). The Securities Commission of Malaysia (SC) requires that P2P lending platforms operating in Malaysia have a minimum paid capital of RM5million, assess and monitor the risk level of each prospective borrower, set limits and obligations, and ensure both issuers and lenders comply with relevant guidelines. SC further established that issuers must be sole proprietorship, partnership, limited liability companies, private unlimited and unlisted companies incorporated in Malaysia. Investors could be any individual or institution outside or within Malaysia, however, depending on rules set by an operator.

### Subscription requirements at P2P lending platforms

As shown in Table 1, information required from potential investors at the nine P2P lending platforms were varied. At each P2P lending platform, potential investors must have initial deposit amounts ranging from RM5 to RM1,000, kept at a specified third-party's account at each platform. Each platform requires each investor to provide basic personal information such as identification card (ID)/passport number, most recent bank account statement, details of sources of income and proof of current residential address. Some P2P lending platforms, such as QuicKash, Money Save, and AlixCo, require additional documents: a selfie photo or a video clip that clearly shows the face of the potential investor while holding his/her passport/ID with the first page open. Most of the platforms allow only Malaysian investors, except QuicKash, MicroLEAP, CapSphere, and AlixCo. Funding Societies, in particular, welcomes investors from four countries, namely Thailand, Indonesia, Singapore, and Malaysia for investment opportunities. Nusa Kapital specifically welcomes Malaysian Muslim investors to participate. Money Save is the only platform that assesses potential investors' financial literacy to categorize them into three groups of investors: (1) with basic investment knowledge, (2) with basic investment knowledge and experience, and (3) with sophisticated investment knowledge and experience.

**Table 1.** Required documents for investors at nine P2P lending platforms in Malaysia.

P2P platforms	Selfie photo/video with face and IC/passport	IC/passport	Bank statement	Utility Bill	Malaysian citizens	Muslim are encouraged	Foreigners	Source of income	Assessment of financial literacy
B2B	No	Yes	Yes	Yes	Yes	No	No	Yes	No
AlixCo	Yes	Yes	Yes	Yes	Yes	No	Yes	Yes	No
Cofundr	No	Yes	Yes	No	No	No	No	Yes	No
Nusa Kapital	No	Yes	Yes	Yes	Yes	No	Yes	Yes	No
CapSphere	No	Yes	Yes	No	Yes	No	No	Yes	No
MicroLeap	No	Yes	Yes	No	Yes	No	Yes	Yes	No
Money Save	Yes	Yes	Yes	No	Yes	No	No	Yes	Yes
Fundaztic	No	Yes	Yes	No	Yes	No	No	Yes	No
QuicKash	Yes	Yes	Yes	No	Yes	No	Yes	Yes	No

### Credit rating and interest rate

Credit rating is one of the most important criteria for most lending institutions, including P2P lending platforms, when deciding on the interest rate that should be charged to a borrower. A credit rating is given to a borrower based on evaluating the potential default incurred by that borrower. In Malaysia, credit ratings can be obtained from Credit Bureau Malaysia. Upon request from a lending institution, Credit Bureau Malaysia provides information such as personal identifying data, personal credit histories reported by various lenders previously, information showing the honesty and stability of a borrower, and the number of requests made by lenders and legal authorities on the borrower's credit status. Credit scores are determined based on factors that differentiate between a good and a bad borrower. A borrower with a good credit rating score can be offered lower interest rates.

However, it is found that some P2P lending platforms in Malaysia use the scores provided by Credit Bureau Malaysia, while others use their own formula to determine the scores for their borrowers. Additionally, the credit ratings are not shared between P2P lending platforms, which leads to varying credit scores across all platforms. It is unknown to investors how these scores are decided, as a result, a borrower may get a low credit score from one P2P lending platform and a higher from another.

**Figure 1.**
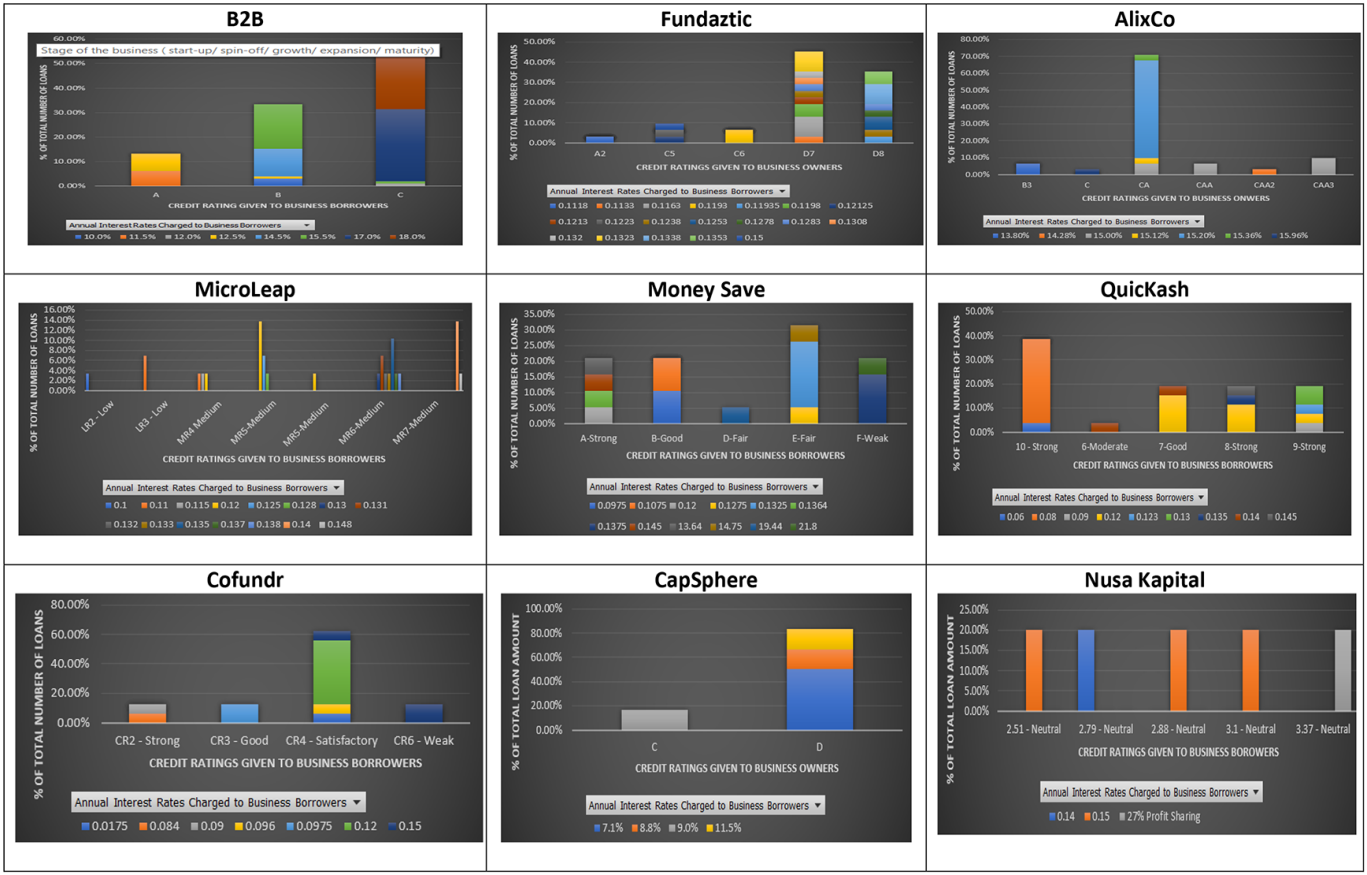
Interest rates and credit ratings at nine P2P lending platforms.

As shown in Figure 1, the principle that loans issued by SMEs with higher credit ratings should be charged with lower interest rates does not hold for most P2P lending platforms, except for MicroLEAP.

The results show that some loans issued by SMEs with lower credit ratings were charged with lower interest rates. For instance, at B2B a loan issued by an SME with a lower credit rating (C), was charged with an interest rate of 12% per annum. In comparison, A was charged with a higher interest rate of 12.5% per annum for a loan issued by an SME with a higher credit rating ( Figure 1). Similarly, at AlixCo platform, a loan issued by an SME with a credit rating of CAA2 (Very high credit risk) (see rating classification at https:/www.alixco.com/fag/statistics) was charged with an interest rate of 14.28% per annum, which is lower than what was (15% per annum) charged for loans issued by SMEs with CAA and CAA3. At Money Save platform, loans issued by SMEs with credit rating A were charged with interest rates ranging from 12% to 14.5% per annum, while loans issued by SMEs with credit rating B were charged with a much lower range of interest rates, i.e., 9.75% - 10.75% per annum.

Similarly, at QuicKash platform, an interest rate of 12% per annum was charged to SMEs with credit ratings ranging from 7-Good to 9-Strong. For Cofundr platform, a loan issued by an SME with a credit rating of CR3-Good was charged with a higher interest rate (9.75% per annum) as compared to that (9.6% per annum) charged to a loan issued by an SME with a lower credit rating (CR4-Satisfactory). Similarly, at CapSphere, loans issued by SMEs with a credit rating D were charged with lower interest rates (7.1%, 8% and 8.8%) as compared to those (9% and 11% per annum) charged to loans issued by SMEs with a credit rating C. Also, at Nusa Kapital platform, an interest rate of 15% per annum was charged to loans issued by SMEs with different credit ratings.

The above findings show that loans with similar credit ratings may offer investors very different interest rates. This result implies that a loan issued by a borrower with a higher credit rating can offer the same or higher interest rate charged to a loan issued by a borrower with a lower credit rating.

### Interest rate and loan purpose

**Figure 2.**
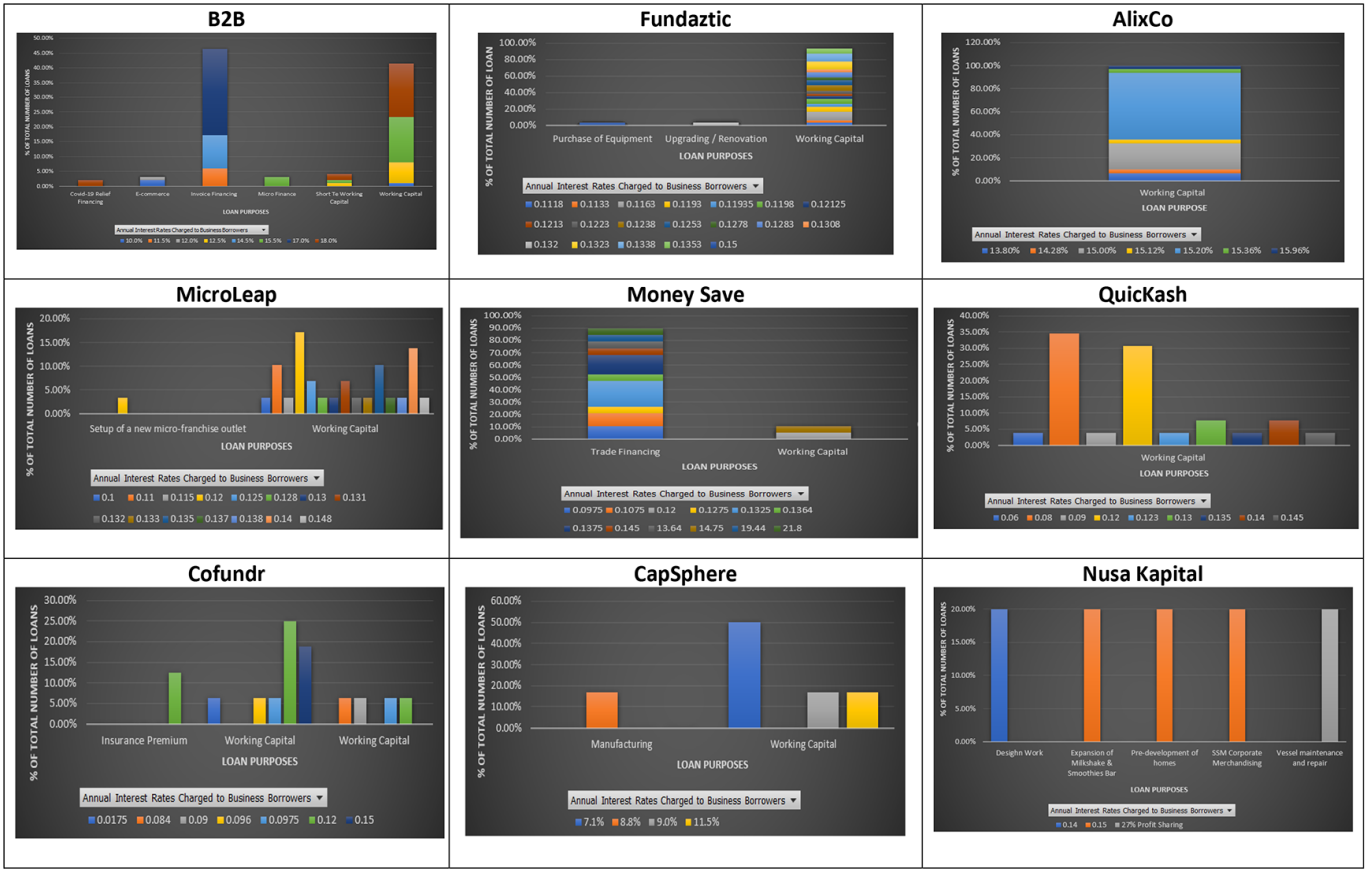
Interest rates and loan purposes at nine P2P lending platforms.

Loan purpose is the main reason for a borrower to request a loan. Lending institutions often assess a borrower's loan purpose to see the level of default risk associated with the requested loan. Regularly stated loan purposes declared at the nine P2P lending platforms are invoice financing, working capital, Covid-19 relief financing, trading, expansion, insurance premium, and equipment maintenance ( Figure 2). As shown in Figure 2 there appears to be no specific interest rate applied to a particular loan purpose at the nine individual platforms. This result may suggest that loan purpose may not be one of the main criteria for a P2P lending platform for determining an interest rate for a borrower. This finding could be due to the similar risk level associated with the previously mentioned loan purposes.

These results show that loans with a similar loan purpose may offer investors significantly different interest rates. In addition, loans with different borrowing purposes can be charged with the same interest rate, suggesting that loan purposes may not indicate much difference in potential risks associated with them at the nine P2P lending platforms.

### Interest rate and loan duration

Duration measures the sensitivity of the value of a debt instrument when the interest rate changes. According to the term structure of interest rates, loans with a longer duration should be charged with a higher interest rate due to the high possibility that interest rates may change during that long period.

**Figure 3.**
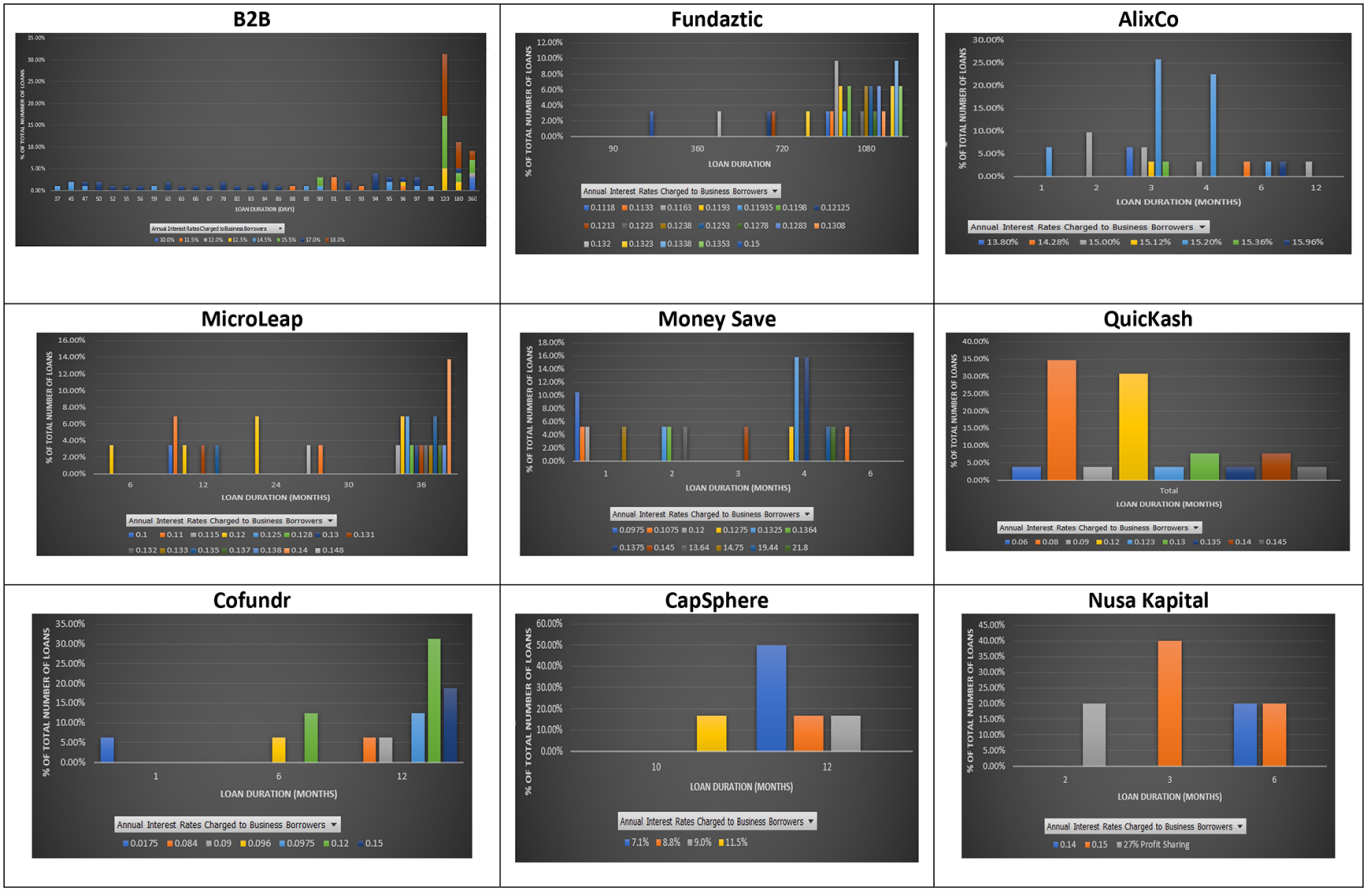
Interest rates and loan durations at nine P2P lending platforms.

However, this does not seem to be the case for the nine P2P lending platforms. As shown in Figure 3, a similar interest rate was applied to loans with different maturities at the nine P2P lending platforms. At B2B FinPal, loans with short durations, i.e., 37 days, had a higher interest rate than those charged for loans with a 98-day duration. Similarly, at Fundaztic, loans with a three-month duration are charged an interest rate of 15%, while a loan with a 36-month duration is charged with a lower interest rate, i.e., 12.23%. At AlixCo, the highest interest rate (15.36%) is charged to a three-month loan, while other loans with four- and six-month durations are only charged at 15%. At MicroLEAP, a 12-month loan is charged with a higher interest rate (14%) than those (12%, 11.5% and 11%) charged to 24-, 30- and 36-month loans. At Money Save, six 1-month loans offer investors interest rates of either 14.75% or 12%, while a six-month loan offers investors a 10.75% interest rate.

At Cofundr, a one-month loan is charged with the highest interest rate of 17.5%. At CapSphere, some 12-month loans are charged with lower interest rates (8.8% per annum) than those (10% per annum) charged to six-month loans. At NursaKapital, a three-month loan is also charged with a higher interest rate (15%) than that (14%) charged to a six-month loan.

These results show that loans with a shorter duration may offer investors higher interest rates than loans with longer durations. This suggests that individual investors can look for loans with a duration of one month and still earn an interest rate that is much higher than the one offered by a 12-month loan at the nine P2P lending platforms.

### Interest rates and business cycles

Most companies asking for loans at the nine P2P lending platforms are in one of the three main stages: growth, extension, and maturity. A stage that a business is going through may indicate the uncertainties that it may face. In a typical business cycle, a company goes through seven stages: seed, startup, growth, established, extension, decline, and exit. There are plenty of opportunities for growing companies; however, they may be exposed to high competition, unstable economy, and market demand, which may hamper their business. Thus, it is also a concern for lenders when granting loans to such businesses. They aim to expand the market share and achieve a new profit level for companies in their extension stages. However, if the expansion to a new product line adds considerable risk to a firm, lenders may need to evaluate loan purposes for such businesses. At a mature/decline stage, companies can experience a drop in their revenues and profits, and therefore might want to close their business. It is due to this reason that lenders find granting loans to these businesses a high risk.

**Figure 4.**
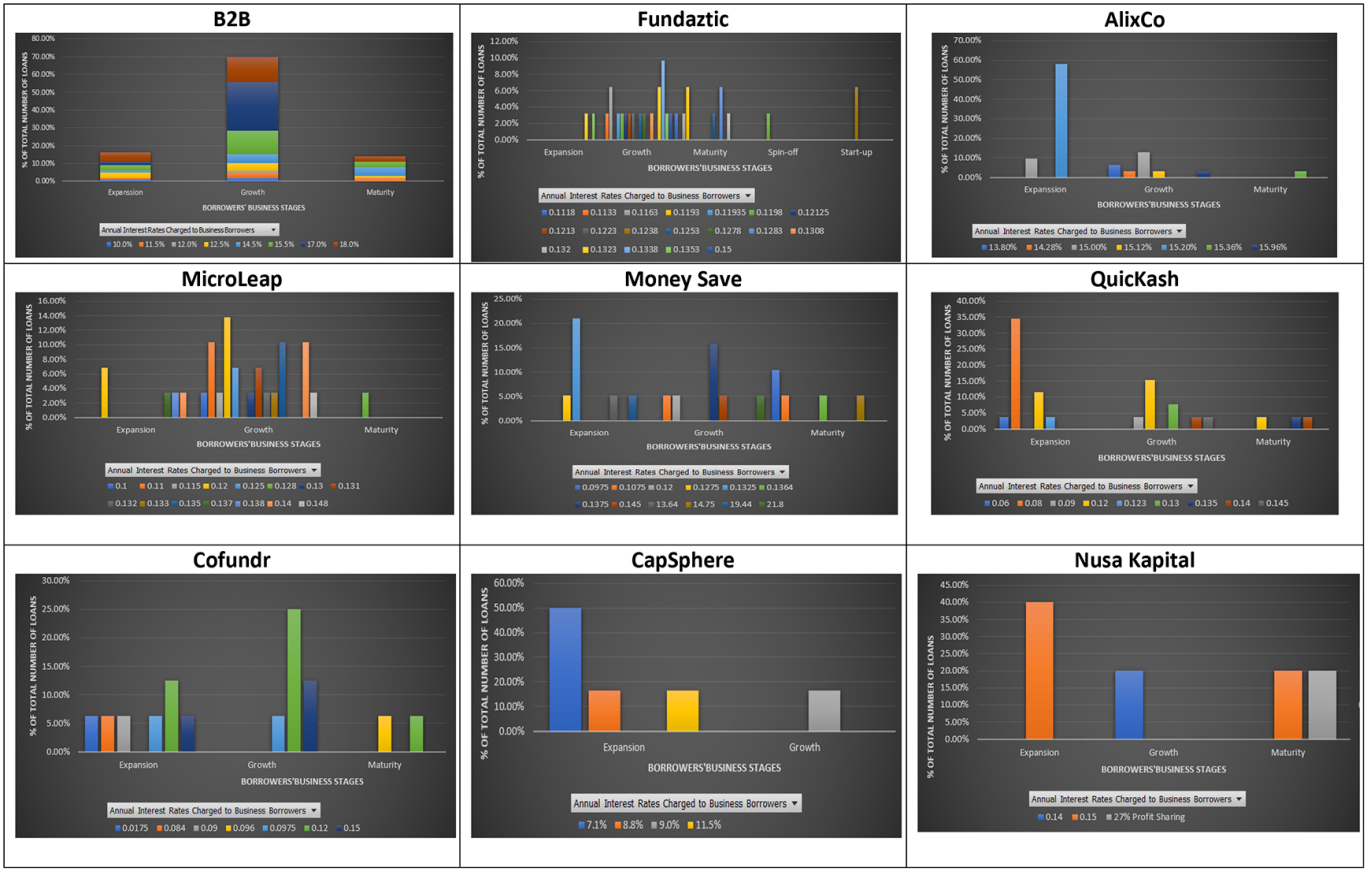
Interest rates and stages of business at nine P2P lending platforms.

As shown in Figure 4, most loans given by the nine P2P lending platforms are for companies in their growth and expansion stages. However, mixed interest rates are charged to both growing and expanding companies at most platforms, except Nusa Kapital. This finding may be mainly due to the limited data available on Nusa Kapital. The stage of a business does not reflect its entire potential risk. A similar interest rate may be charged to both growing and expanding companies on the same platform. In eight out of nine P2P lending platforms, fewer loans are given to mature businesses. There are two possible reasons for this: (1) mature businesses can get loans at traditional lending institutions at lower rates, (2) P2P lending platforms may view small mature businesses much riskier than growing and expanding businesses due to their potential closure.

In short, loans issued by companies are primarily in their growth and expansion stages. It is shown that similar interest rates may be charged to loans issued by companies at different business stages. Moreover, there is no specific range of interest rates applied to loans issued by companies at the same business stage, from the same platform. In other words, the business stage may not be an essential factor in evaluating risks by the nine P2P lending platforms.

### Interest rate and industry

If an industry is less sensitive to any economic downturns, companies from that industry are often expected to have stable businesses for a long time. Thus, lending institutions may prefer to provide loans for such businesses.

**Figure 5.**
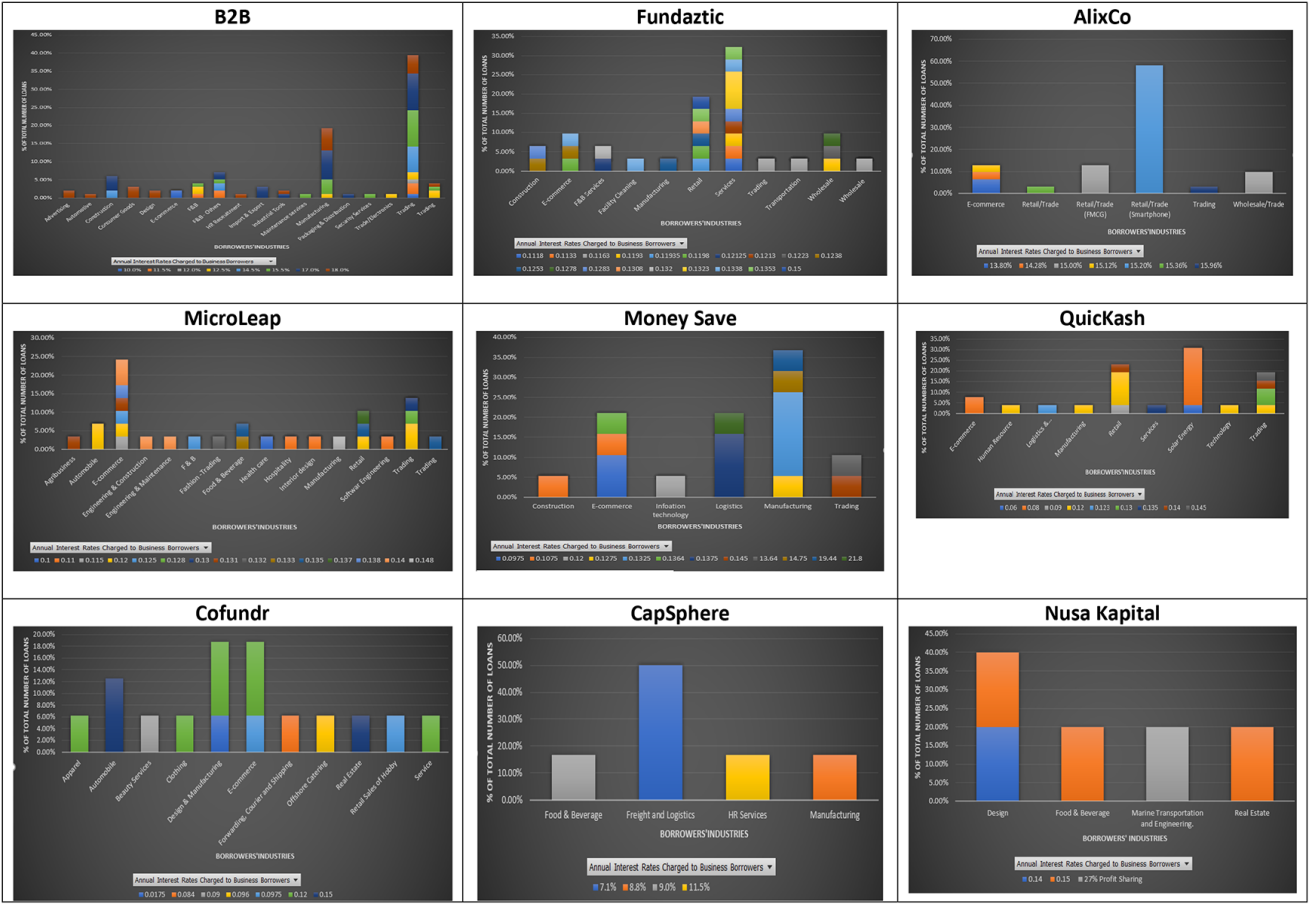
Interest rates and industries at nine P2P lending platforms.

The ununiformed classification of industries in the nine P2P lending platforms, as shown in Figure 5, may indicate potential different risk assessments done by each platform for businesses applying for loans. The results show that at B2B the industries with two or more loans charged with a similar interest rate are: advertising (2), consumer goods (3), design (2), import and export (3), and E-commerce (2). In contrast, loans from the same industry, i.e., construction, food and beverages, packaging and distribution, and trading, can be charged with different interest rates. For AlixCo, loans that come from two classified industries: Retail/Trade (FMCG) (4) and Retail/Trade (Smartphone) (18), are charged at the same interest rates, 14.28% and 15.2%, respectively. However, loans from the E-commerce industry are charged with different interest rates, ranging from 13.8% to 15.1%. For MicroLEAP, two loans from the automobile industry are charged at the same interest rate (12%), while loans from the E-commerce industry are charged with different interest rates, ranging from 11.5% to 14%. For Money Save, it appears that loans from the same industries are charged with different interest rates. For QuicKash, only loans from E-commerce are charged at the same rate of 8% per annum. However, it cannot be determined if all future loans from E-commerce will be charged at the rate of 8% since only two loans were reported for this industry at the time of data collection. For Cofundr, loans from Automobile are charged with the same interest rate of 15%. However, there were only two loans reported for this industry.

For CapSphere, three different loans from Freight and Logistics are charged at the same interest rate of 7.1%, while loans from other industries are charged at various interest rates. Due to limited data on Fundaztic and Nursa Kapital, a conclusion cannot be reached for these two P2P lending platforms.

In short, there seems to be a pattern of having loans issued by companies from the same industry being charged with similar interest rates across the nine P2P lending platforms. Thus, investors may select loans issued by companies from industries that often offer higher interest rates to improve their income streams.

## Study limitation

This study provides a first and original insight into the current P2P lending platforms in Malaysia. However, the analysis was based on a small sample size as it only considered nine P2P lending platforms in this country.

## Conclusion

This study examined nine P2P lending platforms in Malaysia to determine the potential exposures faced by investors through various relationships between interest rates and risk classifying factors such as credit rating, industry, business stage, loan purpose and loan duration. The results of this study showed:

• Loans with similar credit ratings may offer investors significantly different interest rates.
• Loans with similar purposes may offer investors different interest rates.
• Loans with shorter durations may offer investors higher interest rates as compared to those with longer durations.
• No specific range of interest rates was applied to loans issued by companies at the same business cycle.
• Loans issued by companies from the same industry appeared to be charged with similar interest.

The five-month collected data in this study provides a first and original insight into Malaysia's current P2P lending platforms, which will be valuable to potential investors to prepare themselves before making their investments at those platforms.

## Data availability

### Underlying data

Figshare: P2P Lending Platforms in Malaysia: What Do

We Know?

Doi: 10.6084/m9.figshare.14880369 ( Nguyen, 2021).

This project contains the following underlying data:

Data file 1. Dataset_P2P lending in Malaysia What do we know_TIM21109.xlsx

Data are available under the terms of the Creative Commons International “No rights reserved” data waiver (CC BY 4.0).

## Ethical approval

The Research Ethics Committee of Multimedia University approved this research to be conducted. The reference number of this approval is: TTO/REC/EA/123/2021.

## Author contributions

All authors contribute to the data collection. The literature review, research framework and data analysis have been conducted by Nguyen Thi Phuong Lan, Wisdom Kalabeki, and Saravanan Muthaiyah. Cheng-Ming Yu evaluated the findings and discussion of the analysis. Hazik Mohamed gave comments on the practical contribution of this project. Kwan Jing Hui assisted with the data subscription.
