# P2P lending platforms in Malaysia: What do we know?

**DOI:** 10.12688/f1000research.73410.2

**Published:** 2022-04-04

**Authors:** Lan Thi Phuong Nguyen, Wisdom Kalabeke, Saravanan Muthaiyah, Ming Yu Cheng, Kwan Jing Hui, Hazik Mohamed

**Affiliations:** 1 FACULTY OF MANAGEMENT, MULTIMEDIA UNIVERSITY, CYBERJAYA, SELANGOR, 63100, Malaysia; 2 Faculty of Accountancy and Management, Universiti Tunku Abdul Rahman, Bandar Sungai Long Cheras, Kajang, Selangor, 43000, Malaysia; 3 Stella Consulting, Stella Consulting, 60 Paya Lebar Square #07-44,, 409051, Singapore

**Keywords:** P2P lending platforms, default risk, SMEs, individual investors, credit rating, FinTech platforms

## Abstract

**Background** - With the recent evolution of Financial Technology (FinTech), 11 peers to peer (P2P) lending platforms have been regulated by the Securities Commission in Malaysia since 2016. P2P lending platforms offer new investment opportunities to individual investors to earn higher rates on return than what traditional lenders usually provide. However, individual investors may face higher potential risks of default from their borrowers. Therefore, individual investors need to understand the potential exposure to such P2P lending platforms to make an effective investment decision. This study aims to explore the potential risk exposures that individual investors may experience at Malaysia's licensed P2P lending platforms.

**Methods** - Based on data collected manually from nine P2P lending platforms over five months, relationships between interest rates and various risk classifying factors such as credit rating, industry, business stage, loan purpose, and loan duration are examined.

** Results**- This study shows that loans with a similar credit rating and with or without similar loan purpose; and a business stage may offer investors significantly different interest rates. In addition, loans with shorter durations may provide investors with higher interest rates than those with longer durations. Finally, loans issued by companies from the same industry appeared to be charged with similar interest. These findings are valuable to investors to prepare themselves before making their investments at the P2P lending platforms.

**Conclusion**- With first hand-collected data, this study provides an original insight into Malaysia's current P2P lending platforms. Findings obtained for relationships between interest rates and risk classifying factors such as credit rating, industry, business stage, loan purpose and loan duration are valuable to investors of Malaysian P2P lending platforms.

## Introduction

As the most prominent sector in Financial Technology (FinTech), peer-to-peer (P2P) lending platforms use digital communication technology to connect lenders and borrowers online. FinTech platforms like P2P lending are easily accessed by smartphones and the internet for getting unconventional loans. Unlike traditional banks, these lending platforms do not require collateral, the ownership of a bank account, and a business plan. Borrowers are rated and categorized based on their risk profile determined by their provided personal information, including information from their social media sites.

Since the first two P2P lending platforms, named “Zopa” and “Prosper”, were launched in the U.K. and U.S. in 2005 and 2006, respectively, the P2P lending industry has grown and reached Asia. The credit crunch experienced by many traditional banks in the U.S. because of the 2008 Global Financial Crisis, forced many borrowers to turn to P2P lending platforms for their short-term financing needs. Business Wire (2020) reported that the annual compounded growth rate of the global peer to peer (P2P) lending market was around 25% between 2014 and 2019. Although there has been a slowdown in FinTech investments across America and Europe since 2016, the number of P2P lending platforms has increased in many emerging markets in the past few years ( Stern, Makinen, & Qian, 2017).

Since 2016, 11 peer-to-peer (P2P) lending platforms have been granted licenses by the Securities Commission in Malaysia. Since then, the industry has experienced healthy growth in its first two and a half years of operation, as reported by Zhe (2019). Within the first nine months of operation, RM521.7 million were raised by the first six licensed P2P lending platforms to fund 1,560 small and medium enterprises (SMEs). By the end of 2019, the numbers of registered investors at the three most active platforms, Funding Societies Malaysia, Fundaztic and B2B FinPal, were 3,500, 1,700, and 1,500, respectively.

Kok (2021) reposted that many P2P lending platforms, i.e., Funding Societies Malaysia and Fundaztic, experienced steady growth in 2020 despite the Covid-19 pandemic. Their default rates were reported to be reasonably low at 3% and 2.5% per annum by Funding Societies and Fundaztic, respectively. There are two main reasons for this strong growth of P2P lending platforms: the cut in interest rates by Bank Negara Malaysia from 3% to 1.75% between December 2019 and July 2020, and the growth in the use of digital space during the pandemic. Thus, P2P lending platforms appear to be attractive alternative investment platforms as they offer much higher interest rates, ranging from 6% to 21% per annum across the eleven platforms.

Unlike P2P lending platforms in the West, P2P lending platforms in Malaysia are designed to give out loans only to businesses and not individual borrowers. For SMEs that cannot provide sound evidence on their business performance and enough collateral, P2P lending platforms are their best financing alternatives. However, this could imply a possibly higher rate of default faced by the P2P lending industry in Malaysia than traditional lending institutions. Therefore, investors are often advised to diversify their funds across different investment notes while investing at each platform. However, having an effective diversification strategy depends heavily on two critical elements: (1) whether investors have basic financial literacy and (2) how much information of each investment note is made known to investors by each P2P platform.

When it comes to evaluating the default risk of a borrower, information that can help arrive at one’s estimated credit score is needed. For P2P lending platforms, the borrowers’ credit score is based solely on information supplied by the borrower ( Li, 2018). Since the information provided by P2P lending platforms are limited and not guaranteed in the case of Malaysia, it would be even more challenging for investors with good financial literacy to reach a sound investment decision. To our best knowledge, no past studies were carried out with actual loan data collected from P2P lending platforms in Malaysia. Thus, this study will be the first to examine potential exposures that individual investors may experience at licensed P2P lending platforms in Malaysia.

## Literature review

Before making a lending decision to a borrower, a lender often needs to gather various information related to the requested loan and the borrower, such as required interest rate, credit rating, loan amount, loan purpose, loan duration, and the prospect of the borrower’s business, etc. Such information is vital for the lender to make a good lending decision as they hinder the level of uncertainty associated with the borrower’s requested loan.

A borrower’s credit rating is often the first information that a lender would look at when making his/her lending decision ( Yum, Lee & Chae, 2012). In traditional banking practice, a borrowers’ creditworthiness is assessed based on the 5’C: (1) character of the borrower, (2) capital, (3) collateral, (4) capacity and (5) conditions in the market. A credit score will be computed for each borrower. As a result, arriving at a credit rating will determine the interest rate level to be charged to that borrower. A borrower who is given a high credit score will be deemed to have lower default risk and thus will be mostly financed with a lower interest rate, vice versa ( Rivera, 2018; Zhu, 2018). In the P2P lending market, it is unclear how exactly a credit rating is given to a borrower. However, P2P lending platforms often claim that their prospective borrowers’ quantitative and qualitative information will be assessed. Credit scorecard and credit rating algorithms are used together with existing credit scores provided by credit bureaus to arrive at a credit rating for a borrower. For each potential borrower, P2P lending platforms depend mainly on the cashflow statement, potential collaterals that come in any form to secure the loans, and past payment behaviour to estimate a credit score for that borrower ( Suryono, Purwandari & Budi, 2019). Some P2P lending platforms also apply profit scoring approaches since they consider minimising borrowers’ defaults and the intention to maximise lenders’ profits ( Serrano-Cinca & Gutiérrez-Nieto, 2016; Ye, Dong & Ma, 2018). However, such general risk credit scoring methods may be inadequate in providing an accurate probability of default since borrowers from different geographical areas may behave differently ( Ahelegbey, Giudici & Hadji-Misheva, 2018). Thus, moral hazard may be a challenging issue that may keep investors away from the P2P lending industry ( Suryono, Budi & Purwandari, 2020).

It is reported in Zhang, Jia, Diao, Hai and Li (2016) that many AAA-rated borrowers still default in their loans at the PPDai P2P lending platform. Their result suggests that the credit rating given by the platform cannot be used as a predictor for borrowers’ default risks. Some information may be omitted by the P2P lending platform, resulting in a poor default prediction made for a borrower ( Li, Chen & Zeng, 2018; Mi, Hu & Deer, 2018). Therefore, lenders cannot rely on the credit rating given by P2P lending platforms to make their lending decisions alone. Therefore, additional evaluation based on other information about a borrower is necessary for lenders to make sound lending decisions ( Babaei & Bamdad, 2020). Other characteristics of a borrower are essential for lenders to make their investment decisions. For individual borrowers, characteristics such as age, education level, income level, gender, residential location, employment status, etc., may hinder the borrower’s attitude and behaviour towards loan repayment. For business borrowers at P2P lending platforms like the case of Malaysia, characteristics such as types of industry, business cycles, years of establishment, etc., may hinder the level of uncertainty associated with their business income stream, thus, the ability to repay their loans.

A borrower often requests a loan to fulfil a shortage of financial needs over a period, known as loan term or loan duration. A loan with a longer duration appears to have a higher probability of being a default, thus needs to pay a higher interest rate than one with a shorter duration ( Alexander, Guoping, Patrick & Kwabina 2019; Kim, Maeng & Cho, 2020; He, Qin & Zhang, 2021). Therefore, loans with shorter terms are less risky and thus more willingly lent by lenders ( Galak, Small & Stephen, 2011, Prystav, 2016; Ye, Dong & Ma, 2018), although they offer lower interest rates. Loans with longer terms are preferred by risk-loving lenders who seek higher returns ( Zhang, Zhao, Wang & Shen, 2020) or more significant investments ( Tao, Dong & Lin, 2017). In the P2P lending market, lenders prefer loans with shorter loan duration to experience less uncertainty arising from such loans, especially in emerging markets ( Lee & Lee, 2012; Cai, Lin, Xu & Fu, 2016).

Loan purpose is another factor that lenders are interested in before making their lending decisions. If a loan is required to finance less risky financial needs, it is more preferred by the lender ( Serrano-Cinca & Gutiérrez-Nieto, 2016; Xia, Liu & Liu, 2017). Immediate financial needs such as credit card, wedding, automobile, and small home renovation may be perceived by lenders as less risky than longer-term financial needs such as education and business ventures.

At P2P lending platforms, lenders often decide whom to grant the loan primarily based on the interest rate. Therefore, examining the relationship between the prime factor - interest rate and other secondary factors such as credit score, loan term, loan duration, loan purpose, business cycle, and industry will surely provide lenders valuable insights into Malaysian P2P lending platforms before making their investment decisions at those platforms. As such examination has not been done so far for the Malaysian P2P lending platforms, this study attempts to fill in this gap.

## Methods

As P2P lending platforms in Malaysia are designed to give out loans only to businesses, individual borrowers are not eligible to participate in such platforms; however, individual investors can. As P2P lending is open to all Malaysians from different income groups, more individual investors would prefer to subscribe to platforms with affordable subscription fees. Of 11 licensed P2P lending platforms in Malaysia, only ten platforms (Funding Societies, B2B FinPal, Fundaztic, QuicKash, AlixCo, Nusa Kapital, CapSphere, MicroLEAP, Cofundr, and Money Save) require affordable initial deposits, ranging from RM5 to RM1,000, from individual investors. CapBay was excluded from this study because it requires the highest initial deposit (RM10,000) from its investors. Additionally, Funding Societies was also not considered due to the complexity of its subscription. Therefore, the final sample size was nine P2P lending platforms (B2B, Fundaztic, QuicKash, AlixCo, Nusa Kapital, CapSphere, MicroLEAP, Cofundr, and Money Save).

The data collection period was from January to May 2021. Data collection was done manually as a loan campaign is only announced to investors for a maximum of seven days at each platform. Once a campaign is bided on successfully, the issue note will be taken down, and its record will no longer be visible to investors who did not invest in that note. The total sample of loans recorded during the study period consists of 262 loan campaigns captured at the nine Malaysian P2P lending platforms. As shown in Table 1, the B2B platform appears to be the most active platform, with around 99 loan campaigns recorded during the study period, followed by Fundaztic, AlixCo, MicroLEAP and QuicKash. The two least active P2P lending platforms are CapSphere and Nusa Kapital. At some points, the Nusa Kapital platform was not available for investors to participate for unknown reasons. All available information, i.e. loan purpose, loan period, loan amount, credit rating, interest rate, number of payments applied, etc., was obtained from the nine platforms for comparative analysis ( Underlying data) ( Nguyen, 2021).

**Table 1.** Number of loan campaigns recorded from January to May 2021 at the nine Malaysian P2P lending platforms.

Loan amount (RM)	Number of loan campaigns								
	B2B	Fundaztic	Alixco	MicroLEAP	MoneySave	QuicKash	Cofundr	CapSphere	Nusa Kapital
0-10,000	3			7					1
10,001-20,000	2			4			5		
20001-30,000	2	2	1	4				1	1
30,001-40,000	2	1		4	1				
40,0001-50,000	16	3		10		14	1	2	
50,001-60,000	8	2			2		1	1	1
60,001-70,000	3	18			1				
70,001-80,000	4		2		2		1		
80,001-90,000	2				3			1	
90,001-100,000	24	5				9	2		
100,001-110,000	1				4				
110,001-120,000									1
120,001-130,000	2								
130,001-140,000	1				1				
140,001-150,000	4		1		1		2		
150,001-160,000	4				1				
160,001-170,000	1								
170,001-180,000	1					2			
180,001-190,000	1								
190,001-200,000	6		1		2				
200,001-210,000					1				
240,001-250,000			6				1		
270,001-280,000	1								
280,001-290,000									
290,001-300,000	8		4						
300,001-310,000									1
340,001-350,000			4						
380,001-390,000	1								
390,001-400,000			5						
440,001-450,000			3						
450,001-460,000						1			
500,000	1		4						
>500,000	1						3	1	
**Total**	**99**	**31**	**31**	**29**	**19**	**26**	**16**	**6**	**5**

The lender decides whom to grant the loan primarily based on the interest rate. The percentage of the interest rate is mainly dependent on the borrower’s credit score, which is based on various loan characteristics such as the type of business, business cycle, loan purpose, loan duration, and loan amount. Therefore, understanding the relationship between the prime factor - interest rate and other secondary factors such as credit score, loan term, loan duration, loan purpose, business cycle and industry will help investors to be more aware of what to expect when making their investment decisions at those platforms.

### Statistical analysis

Since there is no data publicly available for individual P2P lending platforms in Malaysia, this study employs first hand-collected information for the analysis. Descriptive and cross-tabulation statistics were produced by Microsoft Excel 2010 and IBM SPSS Statistics 26, using a five-month data set to examine the potential exposure that individual investors may face at the nine selected P2P lending platforms. In addition, other tests for normality, coefficients, and multiple regression tests were also conducted. However, the limited number of loan campaigns available during the study period may be insufficient for normality, coefficient and regression tests. Thus, only descriptive statistics are reported and analyzed in this paper as the way to let the raw data speak for itself. This first-hand observation for loan campaigns posted at the nine P2P lending platforms in Malaysia is essential to provide implications to potential investors at those platforms.

## Results/discussion

After the collapse of hundreds of P2P lending platforms in China since 2013 ( Bloomberg Businessweek, October 3, 2018) due to fraudulent activities, P2P lending in Malaysia was somewhat restrictive until 2016. Malaysia is the first country in Southeast Asia to regulate its first six P2P market operators in 2016 (The Securities Commission, 2017). The Securities Commission of Malaysia (SC) requires that P2P lending platforms operating in Malaysia have a minimum paid capital of RM5million, assess and monitor the risk level of each prospective borrower, set limits and obligations, and ensure both issuers and lenders comply with relevant guidelines. SC further established that issuers must be sole proprietorship, partnership, limited liability companies, private unlimited and unlisted companies incorporated in Malaysia. Investors could be any individual or institution outside or within Malaysia, however, depending on rules set by an operator.

### Subscription requirements at P2P lending platforms

As shown in Table 2, information required from potential investors at the nine P2P lending platforms were varied. At each P2P lending platform, potential investors must have initial deposit amounts ranging from RM5 to RM1,000, kept at a specified third-party’s account at each platform. Each platform requires each investor to provide basic personal information such as identification card (ID)/passport number, most recent bank account statement, details of sources of income and proof of current residential address. Some P2P lending platforms, such as QuicKash, Money Save, and AlixCo, require additional documents: a selfie photo or a video clip that clearly shows the face of the potential investor while holding his/her passport/ID with the first page open. Most of the platforms allow only Malaysian investors, except QuicKash, MicroLEAP, CapSphere, and AlixCo. Funding Societies, in particular, welcomes investors from four countries, namely Thailand, Indonesia, Singapore, and Malaysia for investment opportunities. Nusa Kapital specifically welcomes Malaysian Muslim investors to participate. Money Save is the only platform that assesses potential investors’ financial literacy to categorize them into three groups of investors: (1) with basic investment knowledge, (2) with basic investment knowledge and experience, and (3) with sophisticated investment knowledge and experience.

**Table 2.** Required documents for investors at nine P2P lending platforms in Malaysia.

P2P platforms	Selfie photo/video with face and IC/passport	IC/passport	Bank statement	Utility Bill	Malaysian citizens	Muslim are encouraged	Foreigners	Source of income	Assessment of financial literacy
B2B	No	Yes	Yes	Yes	Yes	No	No	Yes	No
AlixCo	Yes	Yes	Yes	Yes	Yes	No	Yes	Yes	No
Cofundr	No	Yes	Yes	No	No	No	No	Yes	No
Nusa Kapital	No	Yes	Yes	Yes	Yes	No	Yes	Yes	No
CapSphere	No	Yes	Yes	No	Yes	No	No	Yes	No
MicroLeap	No	Yes	Yes	No	Yes	No	Yes	Yes	No
Money Save	Yes	Yes	Yes	No	Yes	No	No	Yes	Yes
Fundaztic	No	Yes	Yes	No	Yes	No	No	Yes	No
QuicKash	Yes	Yes	Yes	No	Yes	No	Yes	Yes	No

### Credit rating and interest rate

Credit rating is one of the most important criteria for most lending institutions, including P2P lending platforms, when deciding on the interest rate charged to a borrower. A credit rating is given to a borrower based on evaluating the potential default incurred by that borrower. In Malaysia, credit ratings can be obtained from Credit Bureau Malaysia. Upon request from a lending institution, Credit Bureau Malaysia provides information such as personal identifying data, personal credit histories reported by various lenders previously, information showing the honesty and stability of a borrower, and the number of requests made by lenders and legal authorities on the borrower’s credit status. Credit scores are determined based on factors that differentiate between a good and a bad borrower. A borrower with a good credit rating score can be offered lower interest rates.

However, it is found that some P2P lending platforms in Malaysia use the scores provided by Credit Bureau Malaysia, while others use their own formula to determine the scores for their borrowers. Additionally, the credit ratings are not shared between P2P lending platforms, which leads to varying credit scores across all platforms. It is unknown to investors how these scores are decided. As a result, a borrower may get a low credit score from one P2P lending platform and a higher one from another.

**Figure 1.**
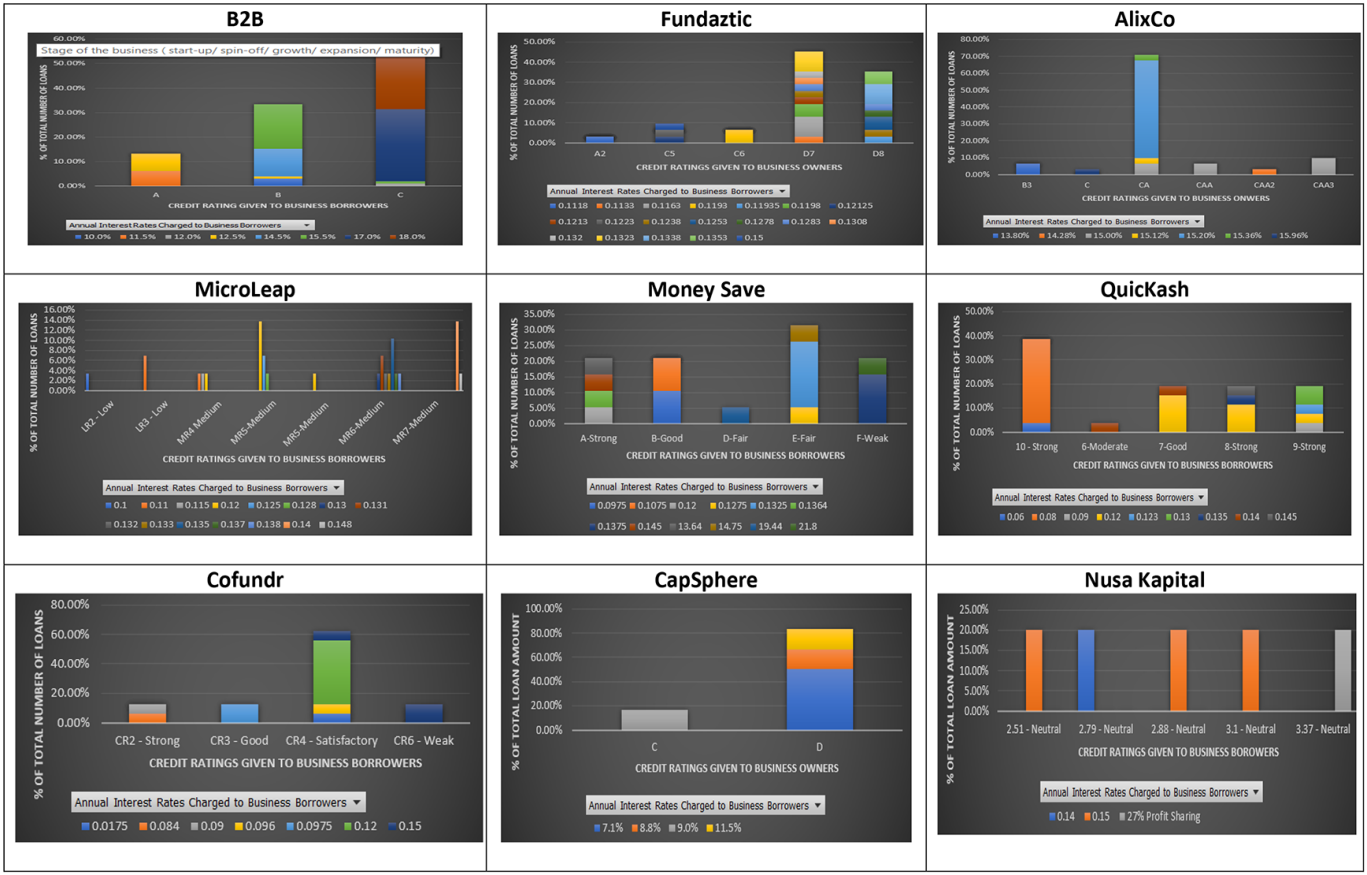
Interest rates and credit ratings at nine P2P lending platforms.

As shown in Figure 1, the principle that loans issued by SMEs with higher credit ratings should be charged with lower interest rates does not hold for most P2P lending platforms, except for MicroLEAP.

The results show that some loans issued by SMEs with lower credit ratings were charged with lower interest rates. For instance, at B2B a loan issued by an SME with a lower credit rating (C), was charged with an interest rate of 12% per annum. In comparison, a loan issued by an SME with an A credit rating was charged with a higher interest rate of 12.5% per annum ( Figure 1). A similar finding is found on the AlixCo platform. A loan issued by an SME with a credit rating of CAA2 (Very high credit risk) (see rating classification at https://www.alixco.com/fag/statistics) was charged with an interest rate of 14.28% per annum, which is lower than what was (15% per annum) charged for loans issued by SMEs with CAA and CAA3 credit ratings. At Money Save platform, loans issued by SMEs with credit rating A were charged with interest rates ranging from 12% to 14.5% per annum, while loans issued by SMEs with credit rating B were charged with a much lower range of interest rates, i.e., 9.75% - 10.75% per annum.

Similarly, at QuicKash platform, an interest rate of 12% per annum was charged to SMEs with credit ratings ranging from 7-Good to 9-Strong. For Cofundr platform, a loan issued by an SME with a credit rating of CR3-Good was charged with a higher interest rate (9.75% per annum) as compared to that (9.6% per annum) charged to a loan issued by an SME with a lower credit rating (CR4-Satisfactory). Similarly, at CapSphere, loans issued by SMEs with a credit rating D were charged with lower interest rates (7.1%, 8% and 8.8%) as compared to those (9% and 11% per annum) charged to loans issued by SMEs with a credit rating C. Also, at Nusa Kapital platform, an interest rate of 15% per annum was charged to loans issued by SMEs with different credit ratings.

The above findings show that loans with similar credit ratings may offer investors different interest rates. This finding suggests that credit ratings given to an SME loan at the Malaysian P2P lending platforms do not reflect the probability of its defaults. Similar to Zhang, Jia, Diao, Hai and Li (2016)’s claim, the meaningless relationship between interest rates and credit ratings found in this study suggest that the credit ratings given by the nine Malaysian P2P lending platform cannot be used as predictors for SMEs’ default risks. The default misrepresenting credit ratings may be due to incomplete information given by the Malaysian SMEs to their respective nine P2P lending platforms, which may lead to a poor prediction on their defaults later, as mentioned by Li, Chen, and Zeng (2018). Furthermore, suppose an SME is given a higher credit rating than it should. Investors attracted to this rating may suffer losses due to potential moral hazards caused by that SME, as also pointed out in Suryono, Budi and Purwandari, (2020). In short, Malaysian investors at the nine P2P lending platforms may enjoy many opportunities when a higher credit-rated SME offers a higher interest rate than a lower credit-rated SME.

### Interest rate and loan purpose

**Figure 2.**
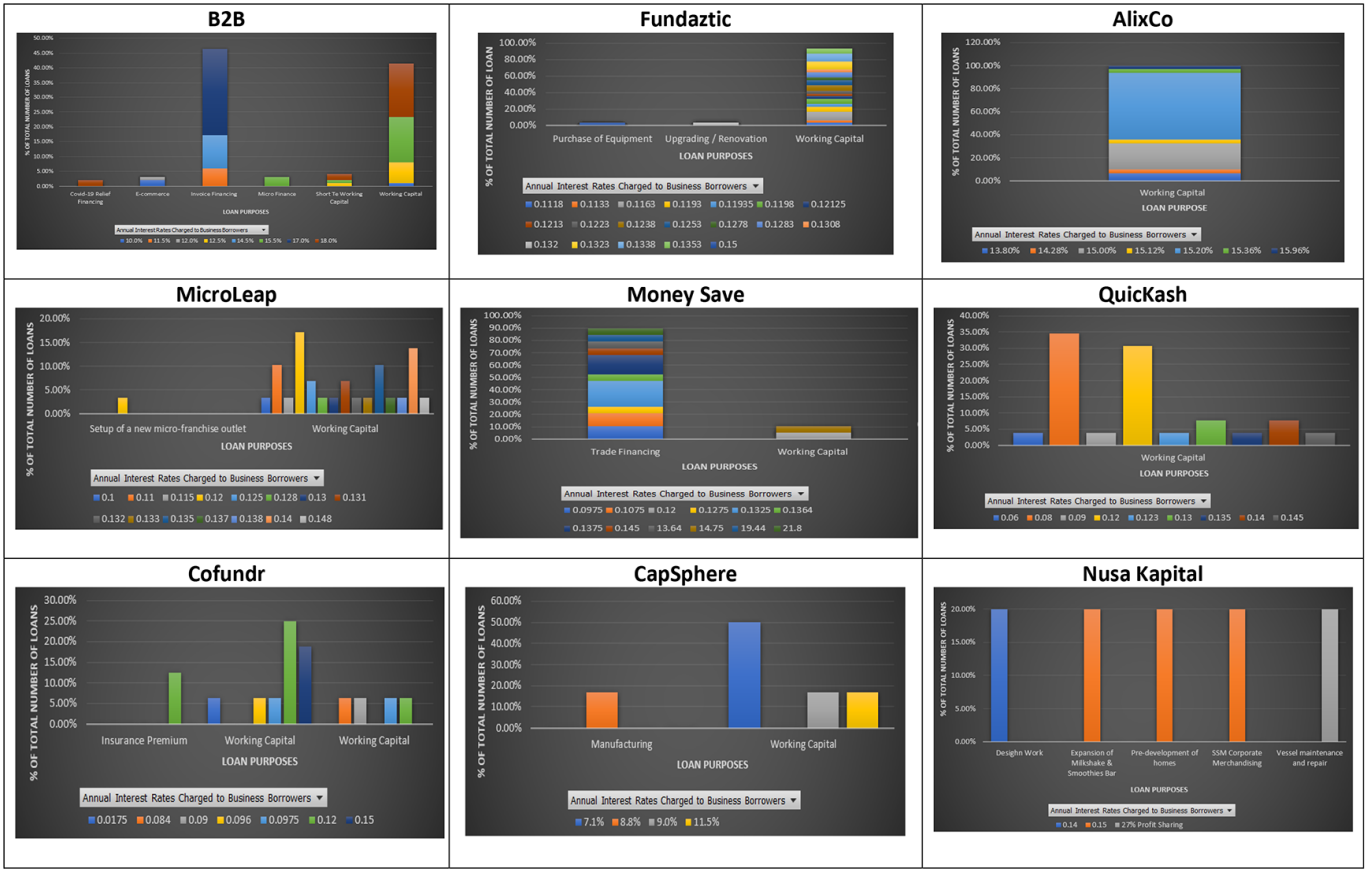
Interest rates and loan purposes at nine P2P lending platforms.

Loan purpose is the main reason for a borrower to request a loan. Lending institutions often assess a borrower’s loan purpose to see the level of default risk associated with the requested loan. Regularly stated loan purposes declared at the nine P2P lending platforms are invoice financing, working capital, Covid-19 relief financing, trading, expansion, insurance premium, and equipment maintenance ( Figure 2). As shown in Figure 2, different interest rates are applied to SMEs whose loan purposes are the same at the nine individual P2P lending platforms. This may suggest that the nine P2P lending forms do not differentiate risk levels among different financial activities associated with their loans. The loan purposes listed at the nine P2P lending platforms mentioned above reflect different risks associated with their financial needs. For instance, a loan applied to cover a temporary shortage of funds, known as invoice financing, due to late payment received from a customer should be viewed as a lower risk loan than a loan applied to expand a business. This is because the uncertainty associated with a business extension in the future is higher than the uncertainty of not getting the payment from a customer within the credit period. This finding implies that a loan required to finance less risky financial needs is not necessarily charged with lower interest rates than those required to finance more risky ones. This way, investors at the nine Malaysian P2P lending platforms can enjoy lending to SMEs with low risky financial needs, as suggested by Serrano-Cinca and Gutiérrez-Nieto (2016) and Xia, Liu and Liu (2017), while still earning good returns. In other words, investing in a low risky loan at the Malaysian P2P lending platforms does not necessarily come with a sacrifice of earning a lower return, as believed.

These results show that loans with a similar loan purpose may offer investors significantly different interest rates. In addition, loans with different borrowing purposes can be charged with the same interest rate, suggesting that loan purposes may not indicate much difference in potential risks associated with them at the nine P2P lending platforms.

### Interest rate and loan duration

Duration measures the sensitivity of the value of a debt instrument when the interest rate changes. According to the term structure of interest rates, loans with a longer duration should be charged with a higher interest rate because interest rates may change during that long period.

**Figure 3.**
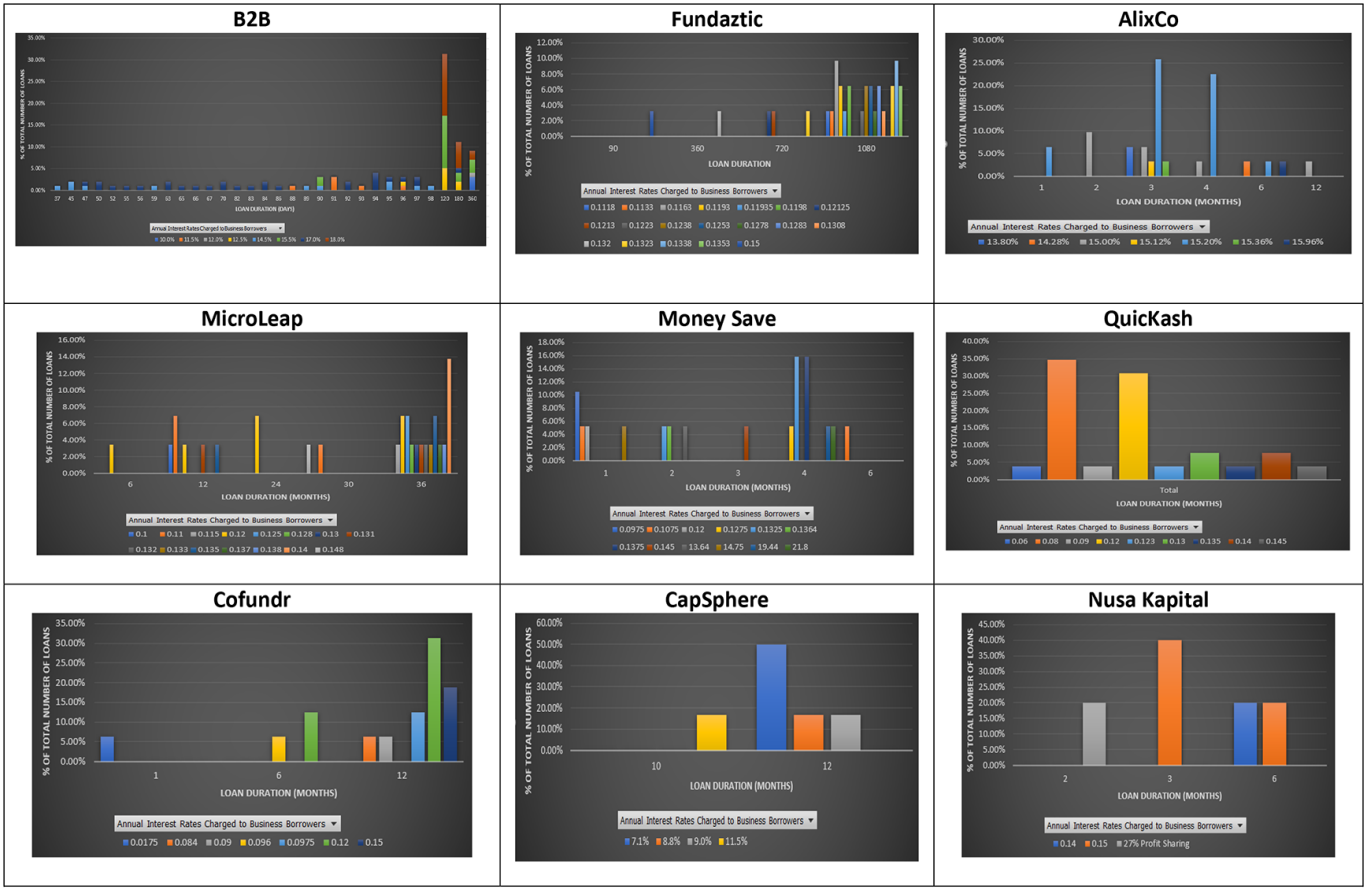
Interest rates and loan durations at nine P2P lending platforms.

However, this does not seem to be the case for the nine P2P lending platforms. As shown in Figure 3, a similar interest rate was applied to loans with different maturities at the nine P2P lending platforms. At B2B FinPal, loans with short durations, i.e., 37 days, had a higher interest rate than those charged for loans with a 98-day duration. Similarly, at Fundaztic, loans with a three-month duration are charged an interest rate of 15%, while a loan with a 36-month duration is charged with a lower interest rate, i.e., 12.23%. At AlixCo, the highest interest rate (15.36%) is charged to a three-month loan, while other loans with four- and six-month durations are only charged at 15%. At MicroLEAP, a 12-month loan is charged with a higher interest rate (14%) than those (12%, 11.5% and 11%) charged to 24-, 30- and 36-month loans. At Money Save, six 1-month loans offer investors interest rates of either 14.75% or 12%, while a six-month loan offers investors a 10.75% interest rate.

At Cofundr, a one-month loan is charged with the highest interest rate of 17.5%. At CapSphere, some 12-month loans are charged with lower interest rates (8.8% per annum) than those (10% per annum) charged to six-month loans. At NursaKapital, a three-month loan is also charged with a higher interest rate (15%) than that (14%) charged to a six-month loan.

The insignificant relationship between loan terms and interest rates suggests that a loan with a longer duration is not necessarily viewed as having a higher probability of default at the nine Malaysian P2P lending platforms. Therefore, it should not be charged with a higher interest rate than one with a shorter duration. Similar to Zhang, Zhao, Wang and Shen (2020)’s finding, investors who invest in longer-term loans may not necessarily earn higher interest rates at the nine P2P lending platforms. In other words, investors at the nine Malaysian P2P lending platforms can avoid high risk by investing in a shorter-term loan as suggested by Galak, Small, and Stephen (2011), Lee and Lee (2012), Prystav (2016), Cai, Lin, Xu and Fu (2016), Ye, Dong and Ma (2018), and still earn higher rates of return.

### Interest rates and business cycles

Most companies asking for loans at the nine P2P lending platforms are in one of the three main stages: growth, extension, and maturity. A stage that a business is going through may indicate the uncertainties that it may face. In a typical business cycle, a company goes through seven stages: seed, startup, growth, established, extension, decline, and exit. There are plenty of opportunities for growing companies; however, they may be exposed to high competition, unstable economy, and market demand, which may hamper their business. Thus, lenders are also concerned when granting loans to such businesses. They aim to expand the market share and achieve a new profit level for companies in their extension stages. However, if the expansion to a new product line adds considerable risk to a firm, lenders may need to evaluate loan purposes for such businesses. At a mature/decline stage, companies can experience a drop in their revenues and profits, and therefore might want to close their business. For this reason, lenders find granting loans to these businesses a high risk.

**Figure 4.**
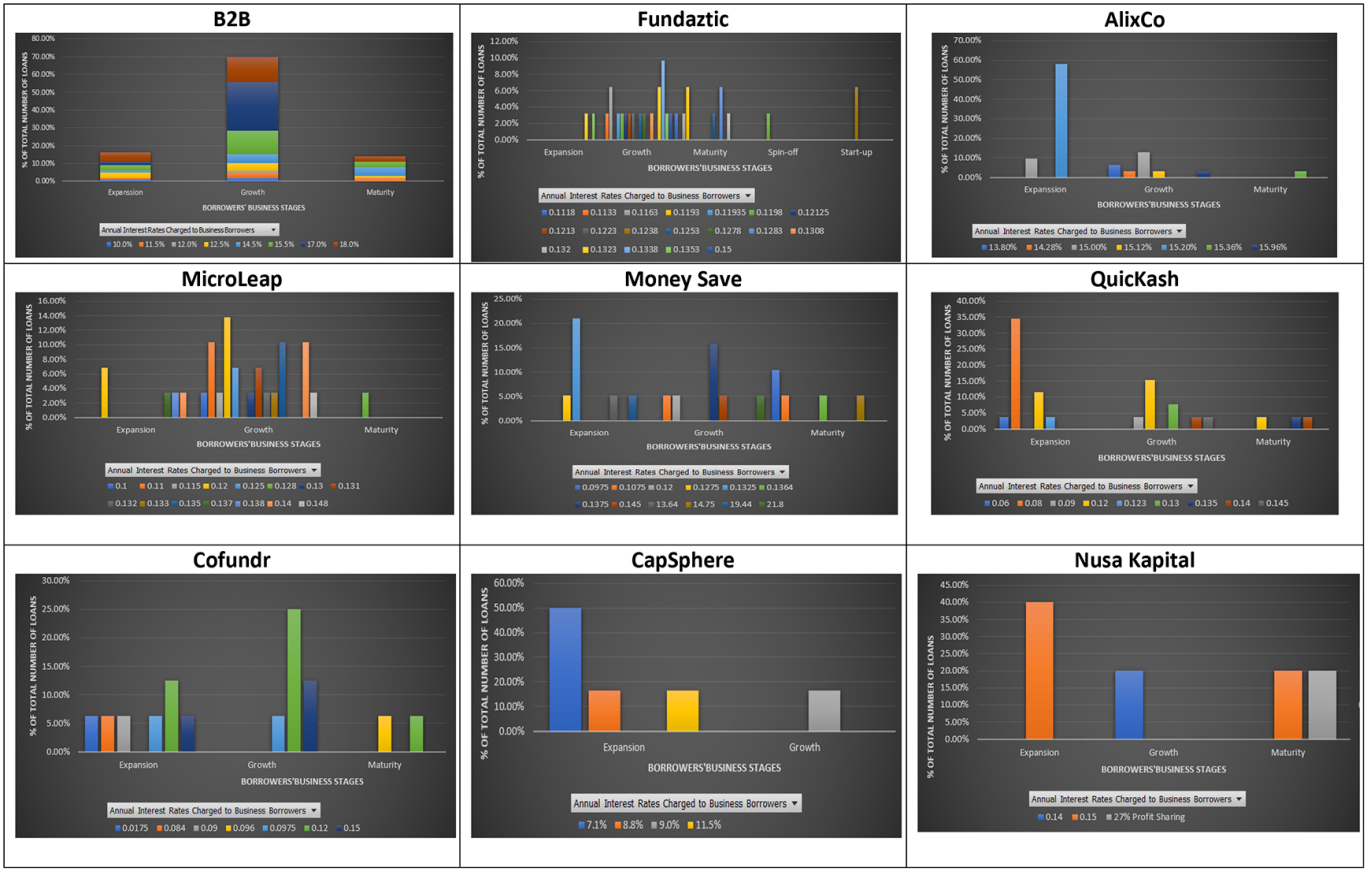
Interest rates and stages of business at nine P2P lending platforms.

As shown in Figure 4, most loans given by the nine P2P lending platforms are for companies in their growth and expansion stages. However, mixed interest rates are charged to both growing and expanding companies at most platforms, except Nusa Kapital. This finding may be mainly due to the limited data available on Nusa Kapital. The stage of a business does not reflect its entire potential risk. A similar interest rate may be charged to both growing and expanding companies on the same platform. In eight out of nine P2P lending platforms, fewer loans are given to mature businesses. There are two possible reasons for this. First, mature businesses can get loans at traditional lending institutions at lower rates. Second, P2P lending platforms may view small mature businesses as riskier than growing and expanding businesses due to their potential closure.

It is shown that similar interest rates may be charged to loans issued by companies at different business stages. In short, loans issued by companies are primarily in their growth and expansion stages. The results suggest that investors who dislike risks may choose to invest in companies with a business cycle with less uncertainty, for instance, expansion stage, and still can enjoy similar or higher interest rates than those in a riskier business cycle, i.e. growth stage, at the nine P2P lending platforms.

### Interest rate and industry

If an industry is less sensitive to any economic downturns, companies from that industry are often expected to have stable businesses for a long time. Thus, lending institutions may prefer to provide loans for such businesses.

**Figure 5.**
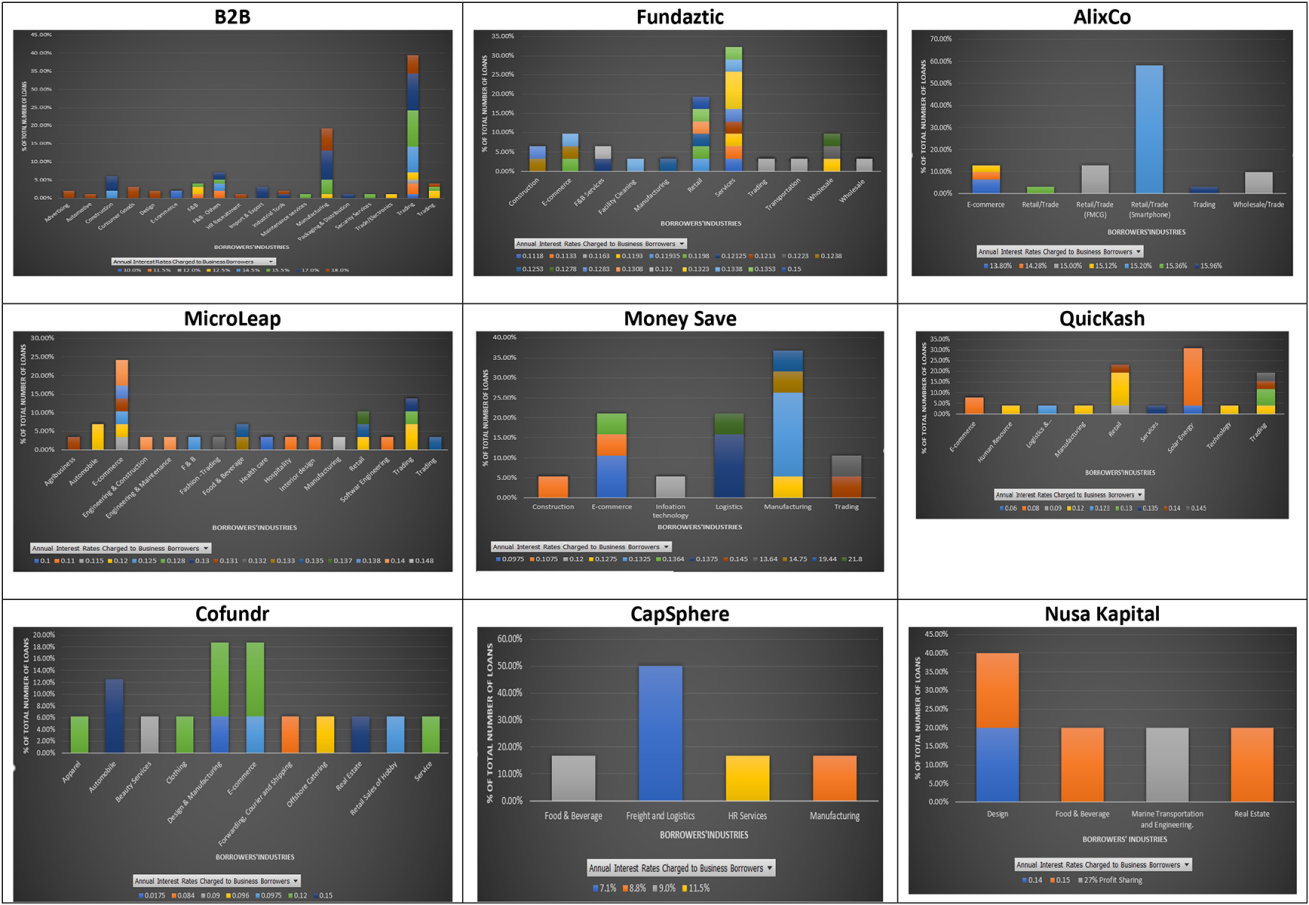
Interest rates and industries at nine P2P lending platforms.

The ununiformed classification of industries in the nine P2P lending platforms, as shown in Figure 5, may indicate potential different risk assessments done by each platform for businesses applying for loans. The results show that at B2B the industries with two or more loans charged with a similar interest rate are: advertising (2), consumer goods (3), design (2), import and export (3), and E-commerce (2). In contrast, loans from the same industry, i.e., construction, food and beverages, packaging and distribution, and trading, can be charged with different interest rates. For AlixCo, loans that come from two classified industries: Retail/Trade (FMCG) (4) and Retail/Trade (Smartphone) (18), are charged at the same interest rates, 14.28% and 15.2%, respectively. However, loans from the E-commerce industry are charged with different interest rates, ranging from 13.8% to 15.1%. For MicroLEAP, two loans from the automobile industry are charged at the same interest rate (12%), while loans from the E-commerce industry are charged with different interest rates, ranging from 11.5% to 14%. For Money Save, it appears that loans from the same industries are charged with different interest rates. For QuicKash, only loans from E-commerce are charged at the same rate of 8% per annum. However, it cannot be determined if all future loans from E-commerce will be charged at the rate of 8% since only two loans were reported for this industry at the time of data collection. For Cofundr, loans from automobile are charged with the same interest rate of 15%. However, there were only two loans reported for this industry.

For CapSphere, three different loans from Freight and Logistics are charged at the same interest rate of 7.1%, while loans from other industries are charged at various interest rates. Due to limited data on Fundaztic and Nursa Kapital, a conclusion cannot be reached for these two P2P lending platforms.

In short, there seems to be a pattern of having loans issued by companies from the same industry being charged with similar interest rates across the nine P2P lending platforms. Also, results show that similar interest rates are applied to companies from different industries. In other words, investors who dislike uncertainty may choose to invest in companies from a less volatile industry, i.e. food/beverage, and enjoy earning similar returns as those from a more volatile industry, i.e. construction.

### Study limitation

This study provides a first and original insight into Malaysia’s current P2P lending platforms. However, the analysis was based on small sample size as it only considered nine P2P lending platforms in this country.

## Conclusion

This study examined nine P2P lending platforms in Malaysia to determine the potential exposures faced by investors through various relationships between interest rates and risk classifying factors such as credit rating, industry, business stage, loan purpose and loan duration. Some worth findings that may be beneficial to investors at the nine Malaysian P2P lending platforms are:

• Loans with higher credit ratings may still offer investors with higher interest rates.
• Loans with less risky financing needs may offer investors higher interest rates.
• Loans with shorter durations may offer investors higher interest rates than those with longer durations.
• Loans with less risky business cycles may offer investors similar or higher interest rates than those with riskier business cycles.
• Loans issued by companies from an industry that experiences less uncertainty may still offer investors a similar interest rate as those from a more volatile industry.

The five-month collected data in this study provides a first and original insight into Malaysia’s current P2P lending platforms, which will be valuable to potential investors to prepare themselves before making their investments at those platforms.

## Data availability

### Underlying data

Figshare: P2P Lending Platforms in Malaysia: What Do

We Know?

Doi: 10.6084/m9.figshare.14880369 ( Nguyen, 2021).

This project contains the following underlying data:

Data file 1. Dataset_P2P lending in Malaysia What do we know_TIM21109.xlsx

Data are available under the terms of the Creative Commons International “No rights reserved” data waiver (CC BY 4.0).

## Ethical approval

The Research Ethics Committee of Multimedia University approved this research to be conducted. The reference number of this approval is: TTO/REC/EA/123/2021.

## Author contributions

All authors contribute to the data collection. The literature review, research framework and data analysis have been conducted by Nguyen Thi Phuong Lan, Wisdom Kalabeki, and Saravanan Muthaiyah. Cheng-Ming Yu evaluated the findings and discussion of the analysis. Hazik Mohamed gave comments on the practical contribution of this project. Kwan Jing Hui assisted with the data subscription.
