# Supplementary material for: P2P lending platforms in Malaysia: What do we know?
Source: F1000Res. 2023 Apr 14;10:1088. Originally published 2021 Oct 26. [Version 3] doi: 10.12688/f1000research.73410.3 (PMC9577281; doi:10.12688/f1000research.73410.3)
Supplement: Peer review history

# Peer review: Reviewer response for version 2

**DOI:** 10.5256/f1000research.122856.r150962

**Published:** 2022-10-17

**Authors:** Rachel J. Nam, Loriana Pelizzon

**Affiliations:** 1 Leibniz Institute SAFE, Goethe University Frankfurt, Frankfurt, Germany

This study compares different SME P2P lending platforms in Malaysia and explores the relationship between credit risk (proxied by interest rates) and different loan characteristics of SME borrowers (loan purpose, duration, business cycle, and industry) using hand-collected data from 9 Malaysian P2P lending platforms regulated by the securities commission.

 Given the low number of loans from these platforms (262 loans from 9 platforms), the authors claim the results from regression tests are inconclusive. Therefore, they provide instead simple descriptive evidence with correlational analyses. This study provides an original insight into Malaysia's current P2P lending platforms. The novelty of the study lies in their first attempt to use loan-level P2P data in Malaysia. One very interesting finding is the disconnect between credit risk (credit scores) and interest rates persistent across many platforms. They find that the credit ratings given by the nine Malaysian P2P lending platforms cannot be used as predictors for SMEs’ default risks. This finding is particularly salient for investor protection purposes since misrepresentation of credit risk can be costly for investors but also for the platform.

 Additionally, they also find that loans with similar loan purposes and durations may also provide investors with different interest rates. Based on these findings, they claim that these factors are not important in explaining potential risks associated with the loans. However, I have reservations with respect to drawing causal inferences using simple univariate comparisons. Without controlling for other factors that may be driving the outcome variable (interest rate), the authors should be cautious of making causal claims.

 These points are further elaborated on below:

  

- Could add further explanations in the literature section as to why credit ratings given by P2P platforms are often *not* reliable? This is contrary to many studies showing the effectiveness of credit scores given by online lenders who leverage alternative data (i.e. Li, Chen & Zeng, 2018; Mi, Hu & Deer, 2018). ^1^^,^^2^ But many studies argue that platform scores using alternative data sources can robustly predict default rates and sometimes outperform traditional credit, For instance, soft information in marketplace lending (Iyer *et al*. 2016), ^3^ friendship, social network, group leader data (Hildebrand *et al*. 2017; Lin *et al.* 2013) ^4^^,^^5^, as well as text-based analysis of applicants listings (Gao *et al*. 2021; Dorfleitner *et al*. 2016; Netzer *et al*. 2019). ^6^^,^{ref-7] ^,^^8^ These studies show non-traditional data used by FinTech lenders improve the predictive power of risk assessment.
- *“For individual borrowers, characteristics such as age, education level, income level, gender, residential location, employment status, etc., may hinder the borrower’s attitude and behaviour towards loan repayment.”*
  - This sentence isn’t so clear. First, what does it mean to “hinder the borrower’s attitude towards loan repayment”? Do you simply mean, these factors are incomplete measures of default probability?
- Figure 1. Credit rating and interest rate: These graphs are too crowded and hard to interpret at first.
- Yet, a very intriguing finding that credit ratings on P2P platforms do not always reflect default probability. Could the authors provide further explanations as to why this may be happening? If credit rating is one of the most important criteria for most lending institutions, why is there such a big disconnect between credit rating and interest rate? Do we know if these credit scores are credit bureau-provided scores or platform-provided scores (credit bureau + alternative data)? The discrepancy is understandable for the former case, but for the latter, a further explanation is needed (i.e. measurement error? Manipulation by the platform? Etc).
- The authors state that “The default misrepresenting credit ratings may be due to incomplete information given by the Malaysian SMEs to their respective nine P2P lending platforms, which may lead to a poor prediction on their defaults later”
  - Can the authors test this theory by looking at the correlation between the interest rate/credit score and how much information was provided by the borrowers on each platform?
  - This still does not explain why there is a misrepresentation of credit ratings. For instance, a firm provides very little information, and gets subsequently charged a higher interest rate. Wouldn’t this also be reflected in the credit score?
- Figure 2. Interest rate and loan purpose: The authors state that “…. different interest rates are applied to SMEs whose loan purposes are the same at the nine individual P2P lending platforms. This may suggest that the nine P2P lending forms do not differentiate risk levels among different financial activities associated with their loans." This is a univariate comparison. Without accounting for other factors that are correlated with loan purpose which may also affect the outcome variable (interest rate), one should be careful in drawing a causal inference. For instance, the level of risk associated with business expansion for a young SME vs. a more established and older SME could be drastically different, which will translate into two different interest rates. This does not imply that P2P platforms do not differentiate risk levels among different loan purposes.
- Figure 3. Interest rates and loan durations: The same concerns apply. Differences in interest rates applied to loans with similar maturities may be driven by other factors. Without controlling for all factors that matter for one’s credit risk, the authors should not draw a causal inference. For instance, the authors claim based on a univariate comparison that “the insignificant relationship between loan terms and interest rates suggests that a loan with a longer duration is not necessarily viewed as having a higher probability of default at the nine Malaysian P2P lending platforms. Therefore, it should not be charged with a higher interest rate than one with a shorter duration.” This statement is simply not true if the loan with a shorter maturity is given to a firm that is inherently riskier in other aspects compared to the firm that is getting a loan with a longer maturity. The positive relationship between loan maturity and risk may still be there if all the other risk factors are controlled for.
- Figure 5: the same comments apply.
- Study limitation: all the analyses are driven by univariate comparisons. This is a correlation study, thus causal interpretations should be interpreted with caution.

Is the work clearly and accurately presented and does it cite the current literature?

Partly

If applicable, is the statistical analysis and its interpretation appropriate?

Partly

Are all the source data underlying the results available to ensure full reproducibility?

Yes

Is the study design appropriate and is the work technically sound?

Partly

Are the conclusions drawn adequately supported by the results?

Partly

Are sufficient details of methods and analysis provided to allow replication by others?

Yes

Reviewer Expertise:

FinTech, crypto and blockchains

We confirm that we have read this submission and believe that we have an appropriate level of expertise to confirm that it is of an acceptable scientific standard, however we have significant reservations, as outlined above.

## References

[1] : The mechanism and effectiveness of credit scoring of P2P lending platform. *China Finance Review International* .2018;8(3) : 10.1108/CFRI-06-2017-0156 256-274 10.1108/CFRI-06-2017-0156

[2] : User Data Can Tell Defaulters in P2P Lending. *Annals of Data Science* .2018;5(1) : 10.1007/s40745-017-0134-z 59-67 10.1007/s40745-017-0134-z

[3] : Screening Peers Softly: Inferring the Quality of Small Borrowers. *Management Science* .2016;62(6) : 10.1287/mnsc.2015.2181 1554-1577 10.1287/mnsc.2015.2181

[4] : Adverse Incentives in Crowdfunding. *Management Science* .2017;63(3) : 10.1287/mnsc.2015.2339 587-608 10.1287/mnsc.2015.2339

[5] : Judging Borrowers by the Company They Keep: Friendship Networks and Information Asymmetry in Online Peer-to-Peer Lending. *Management Science* .2013;59(1) : 10.1287/mnsc.1120.1560 17-35 10.1287/mnsc.1120.1560

[6] : Description-text related soft information in peer-to-peer lending – Evidence from two leading European platforms. *Journal of Banking & Finance* .2016;64: 10.1016/j.jbankfin.2015.11.009 169-187 10.1016/j.jbankfin.2015.11.009

[7] : When Words Sweat: Identifying Signals for Loan Default in the Text of Loan Applications. *Journal of Marketing Research* .2019;56(6) : 10.1177/0022243719852959 960-980 10.1177/0022243719852959

[8] : Linguistic Features and Peer-to-Peer Loan Quality: A Machine Learning Approach. *SSRN Electronic Journal* .2013; 10.2139/ssrn.2446114 10.2139/ssrn.2446114

# Peer review: Reviewer response for version 2

**DOI:** 10.5256/f1000research.122856.r129703

**Published:** 2022-04-11

**Authors:** Ryan Randy Suryono

**Affiliations:** 1 Faculty of Engineering and Computer Science, Universitas Teknokrat Indonesia, Kota Bandar Lampung, Indonesia

I have no further comment.

Is the work clearly and accurately presented and does it cite the current literature?

Partly

If applicable, is the statistical analysis and its interpretation appropriate?

Partly

Are all the source data underlying the results available to ensure full reproducibility?

Partly

Is the study design appropriate and is the work technically sound?

Partly

Are the conclusions drawn adequately supported by the results?

Partly

Are sufficient details of methods and analysis provided to allow replication by others?

Partly

Reviewer Expertise:

Fintech; Financial Technology; P2P Lending; Peer to Peer Lending; Information System

I confirm that I have read this submission and believe that I have an appropriate level of expertise to confirm that it is of an acceptable scientific standard.

# Peer review: Reviewer response for version 1

**DOI:** 10.5256/f1000research.77058.r126134

**Published:** 2022-03-04

**Authors:** Ryan Randy Suryono

**Affiliations:** 1 Faculty of Engineering and Computer Science, Universitas Teknokrat Indonesia, Kota Bandar Lampung, Indonesia

This study explores potential exposures that individual investors may experience at licensed P2P lending platforms in Malaysia.

 1. In the introduction, it is necessary to explain the reason for the the research. Why was this research was conducted? Why Malaysia? This section has very few references.

 2. The methodology section should be explained step by step. What is the process for determining the feasibility of a case study? What is the process of data collection and analysis until it becomes the research result?

 3. The results section should produce an in-depth discussion. How are lessons learned from the findings? Do the results support other research? What are the theoretical and practical implications? Can the author also compare P2P Lending practices in other countries? It might be an interesting review if there are some benchmarks.

 4. Overall, this article needs a lot of improvement in substance by enriching the study literature. Please correct the citation and  proofread.

Is the work clearly and accurately presented and does it cite the current literature?

Partly

If applicable, is the statistical analysis and its interpretation appropriate?

Partly

Are all the source data underlying the results available to ensure full reproducibility?

Partly

Is the study design appropriate and is the work technically sound?

Partly

Are the conclusions drawn adequately supported by the results?

Partly

Are sufficient details of methods and analysis provided to allow replication by others?

Partly

Reviewer Expertise:

Fintech; P2P Lending

I confirm that I have read this submission and believe that I have an appropriate level of expertise to confirm that it is of an acceptable scientific standard, however I have significant reservations, as outlined above.

## References

[1] : Peer to Peer (P2P) Lending Problems and Potential Solutions: A Systematic Literature Review. *Procedia Computer Science* .2019;161: 10.1016/j.procs.2019.11.116 204-214 10.1016/j.procs.2019.11.116

[2] : Challenges and Trends of Financial Technology (Fintech): A Systematic Literature Review. *Information* .2020;11(12) : 10.3390/info11120590 10.3390/info11120590

# Response

**Published:** 2022-03-25

**Authors:** Thi Phuong Lan Nguyen

**Affiliations:** MMU, Malaysia

Dear Prof. Dr. Ryan Randy Suryono,

 First of all, we would like to express our sincere thanks and gratitude for being one of the reviewers for our research paper.

 Upon receiving all your constructed comments, we have revised and made corrections accordingly.

 The following are our responses to your given comments:

 1. For the comment #1: In the introduction, it is necessary to explain the reason for the research. Why was this research was conducted? Why Malaysia? This section has very few references.

 We have added the justification for the study as suggested (see Page 3).

 We have also added a section of literature review to show the related works and the gap in literature the study aims to fill in (see Page 3).

 2. For the comment #2: The methodology section should be explained step by step. What is the process for determining the feasibility of a case study? What is the process of data collection and analysis until it becomes the research result?

 We have added details of the sample of loan campaigns recorded from the nine P2P lending platforms (page 8).

 The following table is added:

 Table 1: Number of Loan Campaigns Recorded from January to May 2021 at the Nine Malaysian P2P Lending Platforms (Page 9)

 We have also explained the analysis method used for the study (Page 8).

 3. For the comment #3: The results section should produce an in-depth discussion. How are lessons learned from the findings? Do the results support other research? What are the theoretical and practical implications? Can the author also compare P2P Lending practices in other countries? It might be an interesting review if there are some benchmarks.

 We have added more interpretation of the results, linked them to past studies and practices, and provided implications and suggestions to each finding section. (see Page 9 - Page 14)

 4. For the comment #4: Overall, this article needs a lot of improvement in substance by enriching the study literature. Please correct the citation and proofread.

 We have also added a section of literature review to show the related works and the gap in literature the study aims to fill in (see Page 3).

 We have added more references and corrected citations as suggested. 

 The article has gone through a few rounds of proofreading. (see page 15 – 16)

 We are looking forward to having further feedback and approval.

 Many thanks,

 On behalf of the team,

 Lan Nguyen (Ph.D.)

# Response

**Published:** 2022-04-05

**Authors:** Ryan Randy Suryono

**Affiliations:** Universitas Teknokrat Indonesia, Indonesia

All suggestions for revision have been done well. Authors have added more interpretations of the results, linked them to previous studies and practice, and provided implications and suggestions for each section of the findings listed on Page 9 - Page 14.

 Suggestions for further research, conducting a qualitative approach/empirical study, can also conduct Focus Group Discussions with the P2P Lending platform in Malaysia and capture the P2P Lending landscape. Propose ideas for the industry.

 Good Luck!

# Peer review: Reviewer response for version 1

**DOI:** 10.5256/f1000research.77058.r98013

**Published:** 2021-11-10

**Authors:** Dongwoo Kim

**Affiliations:** 1 Research Institute, KB Financial Group Inc, Seoul, South Korea

This study examined nine P2P lending platforms in Malaysia to determine the potential exposures faced by investors through various relationships between interest rates and risk classifying factors such as credit rating, industry, business stage, loan purpose and loan duration. This paper is meaningful in clarifying the relationship between interest rates and other information on multiple P2P lending platforms based in Malaysia for the first time. However, there are some issues that the author should address:

 1. Citation: some of the references are not displayed in the body text.

 2. Samples: it is necessary to clarify the number of samples for each platform to be analyzed.

 3. Methods: it is necessary to clarify the reason for omitting the results of regression analysis and normality analysis as well as containing only the contents of descriptive statistics.

 4. P2P lending is a field that has recently received a lot of attention academically and practically. Therefore, more and more related studies are being published. Among them, there are several studies that have revealed the relationship between various types of borrower information and loan interest rates, which are of interest to the authors, and should be investigated by them.

Is the work clearly and accurately presented and does it cite the current literature?

Partly

If applicable, is the statistical analysis and its interpretation appropriate?

Partly

Are all the source data underlying the results available to ensure full reproducibility?

Yes

Is the study design appropriate and is the work technically sound?

Yes

Are the conclusions drawn adequately supported by the results?

Yes

Are sufficient details of methods and analysis provided to allow replication by others?

Yes

Reviewer Expertise:

P2P lending

I confirm that I have read this submission and believe that I have an appropriate level of expertise to confirm that it is of an acceptable scientific standard, however I have significant reservations, as outlined above.

# Response

**Published:** 2022-03-25

**Authors:** Thi Phuong Lan Nguyen

**Affiliations:** MMU, Malaysia

Dear Prof. Dr. Dongwoo Kim,

 First of all, we would like to express our sincere thanks and gratitude for being one of the reviewers for our research paper.

 Upon receiving all your constructed comments, we have revised and made corrections accordingly.

 The following are our responses to your given comments:

 1. For the comment #1: Citation: some of the references are not displayed in the body text.

 We have added more references and corrected citations as suggested. 

 2. For the comment #2: Samples: it is necessary to clarify the number of samples for each platform to be analyzed.

 We have added details of the sample of loan campaigns recorded from the nine P2P lending platforms (page 8).

 The following table is added:

 Table 1: Number of Loan Campaigns Recorded from January to May 2021 at the Nine Malaysian P2P Lending Platforms (Page 9)

 3. For the comment #3: Methods: it is necessary to clarify the reason for omitting the results of regression analysis and normality analysis as well as containing only the contents of descriptive statistics.

 We have also explained the analysis method used for the study (Page 8).

 4.  For the comment #4: P2P lending is a field that has recently received a lot of attention academically and practically. Therefore, more and more related studies are being published. Among them, there are several studies that have revealed the relationship between various types of borrower information and loan interest rates, which are of interest to the authors, and should be investigated by them.

 We have also added a section of literature review to show the related works and the gap in literature the study aims to fill in (see Page 3).

 We are looking forward to having further feedback and approval.

 Many thanks,

 On behalf of the team,

 Lan Nguyen (Ph.D.)
